# Autogenerating a Domain-Specific Question-Answering Data Set to Enable High-Performing Language Models for Magnetic Materials

**DOI:** 10.1021/acs.jcim.6c00136

**Published:** 2026-08-27

**Authors:** Charles R. Kelly, Jacqueline M. Cole

**Affiliations:** Ray Dolby Centre, Cavendish Laboratory, Department of Physics, 2152 University of Cambridge, J. J. Thomson Avenue, Cambridge CB3 0US, U.K.

## Abstract

Domain-specific language models are attractive because they can be tailored to suit their users. However, the high-end computational resources needed to pretrain large language models (LLMs) by conventional methods tend to prohibit their development. Furthermore, there is a dearth of large, domain-specific, question-answering (QA) data sets to finetune LLMs for prompt engineering, even when computational resources can be found to pretrain LLMs. Our paper presents a method for autogenerating a data set of 168,080 domain-specific QA pairs relating to magnetic materials. We show how this can be employed to finetune LLMs for the downstream task of Question-Answering (QA) for the magnetic materials domain. We use the bidirectional encoder representations from transformers (BERT) architecture as our benchmark LLM to compare the relative performance of 6 LLMs. These include 3 vanilla (BERT-base-cased) LLMs that have been finetuned on various QA data sets (i) our domain-specific QA data set (MagQA); (ii) the Stanford Question-Answering Data set (SQuAD v2); (iii) our QA data set mixed with SQuAD v2. The other 3 (BERT-base-cased) LLMs have been subjected to domain-adaptive pretraining on a corpus of 97,308 magnetic papers prior to their finetuning on the same combination of QA data sets (i)-(iii). As well as our QA data set autogeneration pipeline, we show how our work can be extrapolated to the larger architectures of RoBERTa-base and DeBERTa-base. We also undertake an ablation study of the finetuning process using four sizes of QA data sets to gain insights into the quantity of data that are needed for sufficient domain-specification. The highest performing LLM was MagBERT_MagQA_Mixed, a vanilla BERT-base-cased LLM that had been finetuned on the combination of our autogenerated MagQA data set and SQuAD v2, achieving an *F*1 score of 78.43% and an exact-match score of 72.84% when tested on a manually annotated data set on magnetic materials. These results demonstrate that domain-specific BERT models only need to be finetuned from vanilla BERT models (*i.e*. no domain-adaptive pretraining is needed), pending the availability of sufficiently large, high-quality, domain-specific QA data sets that this work shows how to autogenerate.

## Graphical abstract

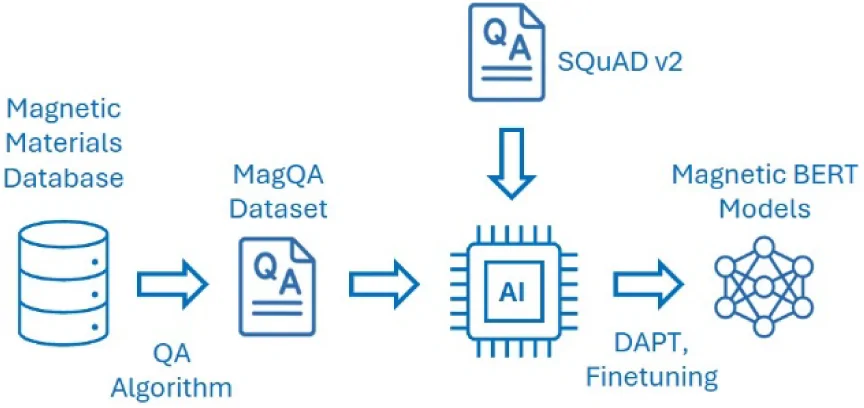

## Introduction

Large language models (LLMs) for the materials-science domain are most typically developed by training them on a corpus of domain-specific published articles as a data set. Vanilla LLM architectures are either further pretrained on this corpus via domain-adaptive pretraining (DAPT) or are trained from scratch^1–8^ using this corpus. Either way, this targeted pretraining method enables the weights of the neural network within the LLM to be adjusted sufficiently in a way that its performance aligns to a specific domain, when subjected to downstream tasks such as question-answering (QA)^9^ that is the basis of prompt engineering.

The provision of domain-adapted LLMs for QA tasks is desirable to end users in materials science owing to their demonstrated capabilities in the data extraction of material-property relationships.^10–13^ Thereby, the extraction of material-property relationships from literature can be framed as a multiturn QA problem.^14^ For example, for a given context, the first-turn question maybe

Example question: “What is the material in the context given?”

Example answer: Iron

This allows for a second-turn question to be asked that is conditional upon the first one, e.g.:

Question: “What is the Curie temperature of the material, <answer to the first turn question>?”

Example answer: 1043 K

Researchers then use these material-property pairs to find structure–property relationships that can be incorporated into a data-driven pipeline that accelerates novel materials design and discovery. A range of domain-specific LLMs for materials science have been pretrained and fine-tuned on QA tasks.^10–13^ However, many of these LLMs have been finetuned on either: generic English language using interactive human prompting, or data sets of QA pairs from generic English language, such as the Stanford Question Answering Data set (SQuAD ∼100,000 pairs).^15^ As such, they do not operate with a high level of domain specificity.

One method of imparting a greater extent of domain-specific knowledge into LLMs is domain-adaptive pretraining (DAPT).^16^ DAPT methods involve vanilla LLMs being further pretrained on a domain-specific corpus. These LLMs are then often finetuned using a data set consisting of a large number of generic QA pairings *as well as* a manually curated data set of domain-specific QA pairings; albeit, this latter data set is usually small owing to the manual nature of its curation. DAPT-generated LLMs are able to understand their target domain and successfully extract material-property pairs from literature.^1–5^ However, DAPT is not without its problems. Further pretraining of vanilla LLMs requires significant compute time and resources, often necessitating multiple hours of training across multiple compute nodes to obtain LLMs which exhibit sufficiently good performance to serve their purpose. In addition, the manual curation of domain-specific QA data sets for finetuning a DAPT-generated LLM to QA tasks can be very time-consuming. The result of these factors can have significant impacts on environmental and financial resources.

Recent work has addressed these issues with DAPT by providing an alternative means to produce domain-specific LLMs. These studies have shown that the CNER pipeline within ChemDataExtractor^17–19^ can be combined with specialized algorithms,^14,20^ to autogenerate large domain-specific QA data sets. These large domain-specific data sets can then be used to finetune domain-specific LLMs for the downstream task of QA and achieve similar or even better results to LLMs that have been trained using DAPT methods. This indicates that there is no longer a need to further pretrain vanilla LLMs to create high-quality domain-specific LLMs.

This study presents an algorithm that creates a large domain-specific QA data set (MagQA) that we autogenerate from a magnetic materials database that contains material-property relationships. We further demonstrate that this MagQA data set can be used for finetuning vanilla LLMs for the downstream tasks of QA with high performance. For comparative purposes, we present 6 LLMs that are all based on the Bidirectional Encoder Representations from Transformer (BERT) architecture^21^ as this serves as a benchmark for performance evaluations in materials science. We compared the performance of 3 BERT models that had been subjected to domain-adaptive pretraining on a large corpus of papers in the magnetic domain, which were subsequently finetuned on three different QA data sets: (i) our MagQA data set; (ii) the generic English language QA data set, SQuAD v2; (iii) a combined set of MagQA and SQuAD v2. The other 3 BERT models were not subjected to any pretraining but were simply finetuned on one of these three QA data set variations, (i)-(iii). A summary of the 6 BERT models, and the data sets that were used to train or finetune them is given in Table 1. We shall demonstrate that domain-specific BERT models that have been finetuned on QA tasks can be produced with top performance using only this finetuning process, subject to the availability of a large, high-quality, domain-specific QA data set which we show can be autogenerated via the methods presented in this work. This has far-reaching consequences because it means that computationally intensive pretraining or further training processes that are typically used to train domain-specific LLMs may be less instrumental and could help pave the way to democratize LLM developments for materials science.

**Table 1:** A Summary of Our 6 BERT Models, and the Additional Datasets We Trained Them on

Model	Starting Model	DAPT on corpus of magnetism papers	Finetuned on our MagQA data set	Finetuned on SQuAD v2
BERT_SQuADv2	BERT-base-cased	No	No	Yes
MagBERT_DAPT_SQuADv2	BERT-base-cased	Yes	No	Yes
MagBERT_DAPT_MagQA	BERT-base-cased	Yes	Yes	No
MagBERT_DAPT_MagQA_Mixed	BERT-base-cased	Yes	Yes	Yes
MagBERT_MagQA	BERT-base-cased	No	Yes	No
MagBERT_MagQA_Mixed	BERT-base-cased	No	Yes	Yes

## Methods

### System Overview

The outline of the operational workflow used in this study is given in Figure 1.

**Figure 1:**
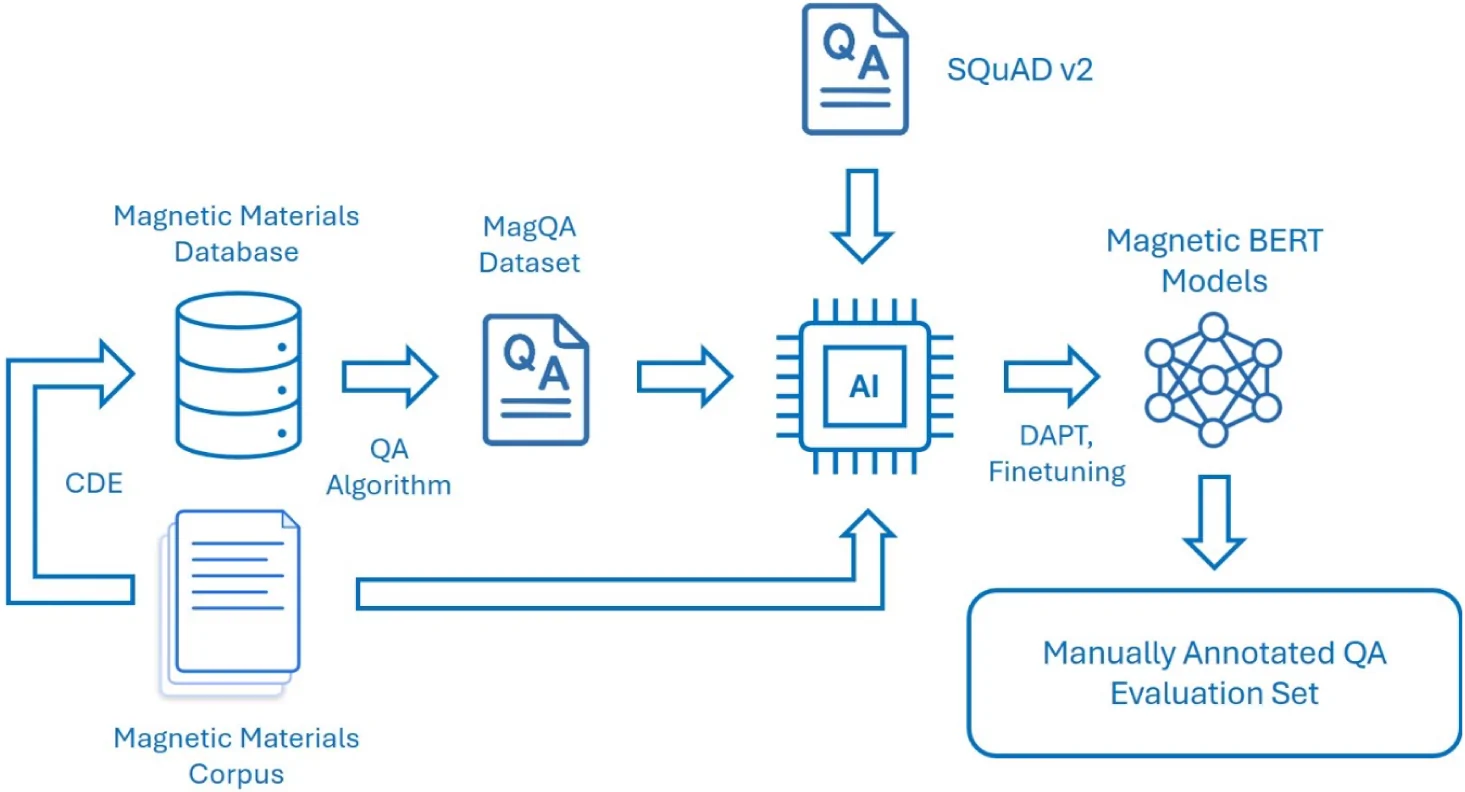
MagBERT workflow presented in this study. Abbreviations used for visual clarity denote Artificial Intelligence (AI), Bidirectional Encoder Representations from Transformers (BERT), ChemDataExtractor (CDE), Domain-Adaptive Pretraining (DAPT), Question-Answering (QA), our magnetic QA data set (MagQA) developed herein and the Stanford Question-Answering Data set, version 2 (SQuAD v2) that contains QA pairs that have been constructed from generic English language.

### Choice of BERT Model Architecture

In the context of BERT-base language models, studies have conclusively shown that a BERT-base-cased model will perform better with masked language modeling (MLM) and the downstream QA task in comparison to its uncased counterpart.^1,3–5^ This is due to the differentiation of cased and uncased tokens, where the former adds more unique information into the model. Therefore, we opted to employ the publicly available BERT-base-cased pretrained language model^21^ for this study. The parameters of the BERT-base-cased model are shown in Table 2.

**Table 2:** Parameters of the BERT-Base-Cased Language Model

Parameter	Value
Number of hidden layers	12
Hidden size	768
Number of attention heads	12
Total number of parameters	∼110 million

### Domain Adaptive Pretraining (DAPT)

A BERT-base-cased language model was further trained using DAPT methods and employing a large corpus of 108,120 academic papers that contain domain-specific data about magnetic materials and their cognate Curie and Néel temperatures. This corpus was collated using web scrapers with targeted queries to mine academic papers in the magnetic materials domain from Elsevier and Royal Society of Chemistry (RSC) publishing houses. The corpus was scraped using the queries “Curie temperature” and “Néel temperature”, which were stored as .xml (Elsevier) and .html (RSC) files. Facilities built into the ChemDataExtractor toolkit^17–19^ were then used to convert these documents into plain text files, and then each text file was combined into a JSON data set with a train and validation split ratio of 90%/10%, resulting in a train split of 97,308 articles and a validation split of 10,812 articles.

Once the domain-specific data set of paper text has been curated and formatted from the original corpus, the publicly available HuggingFace Transformers^22^ implementation of the BERT-base-cased checkpoint was further pretrained on the ALCF Polaris supercomputer to produce a MagBERT-base-cased model using the domain-specific train split of the JSON data set. Overall, the BERT model was trained on the training split of the JSON data set for a total of 21,500 steps, which equated to roughly 38 epochs, and was evaluated using the validation split of the JSON data set every 100 steps; it achieved a final loss of 1.32, an evaluation loss of 1.24, and an evaluation accuracy of 73%. The loss, evaluation loss and evaluation accuracy of the BERT model were captured throughout the DAPT process, as shown in Figure 2.

**Figure 2:**
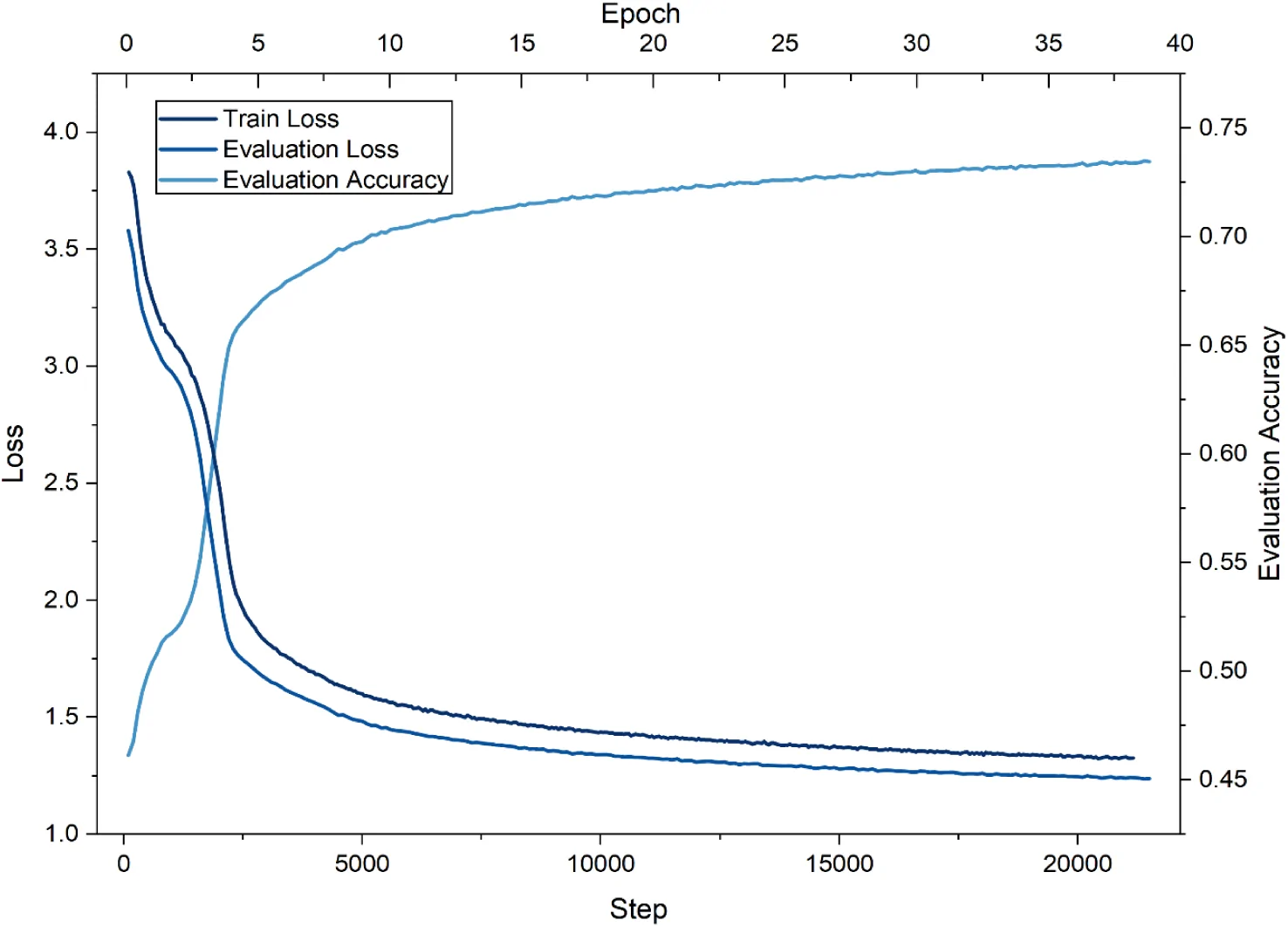
A graph showing steps and epochs against training loss, evaluation loss and evaluation accuracy for the DAPT-generated MagBERT-base-cased model.

The training process was distributed with DeepSpeed,^23^ using 40 Nvidia A100 GPUs spanning 10 nodes and took roughly 875 node hours. The key hyperparameters used for the DAPT of the model are given in Table 3.

**Table 3:** Hyperparameters Used for the DAPT-Generated MagBERT-Base-Cased Model

Hyperparameter	Value
learning rate	1e-5
adam_beta2	0.98
adam_epsilon	1e-8
max_seq_length	512
per_device_train_batch_size	64
warmup_ratio	0.06
weight_decay	0.01
Seed	50

### Fine-Tuning for QA Tasks

The available HuggingFace checkpoint for the BERT-base-cased model has been trained in the tasks of Masked Language Modeling (MLM) and Next Sentence Prediction (NSP). For our BERT models to be suitable for the downstream task of QA, transfer learning is required. Thereby, this study necessitated the replacement of the layer at the end of the neural network within each BERT model, and then all layers were fine-tuned for QA tasks, *i.e*., our BERT models were finetuned according to the HuggingFace implementation of finetuning for QA. During this finetuning process, each new BERT model takes a context and a question as the input and then returns a prediction for the span of the answer within the context as the output. One or both of two QA data sets were used in this fine-tuning process: the Stanford Question Answering Data set (SQuAD) v2 ^15,24^ and our MagQA data set generated via this study. SQuAD v2 provides roughly ∼150,000 QA pairs across its train and validation splits, ∼50,000 of which are unanswerable questions. Each entry in the data set contains a context, taken from English Wikipedia; a question, and if the question is answerable; an answer and the start location of the answer. The unanswerable question concept is new feature to SQuAD v2 that is not in its version 1 predecessor, adding approximately 50,000 unanswerable questions to its data set, i.e., creating an approximate ratio of 2:1 answerable to unanswerable questions. The content of an example record in the SQuAD v2 data set is shown in Figure 3.

**Figure 3:**
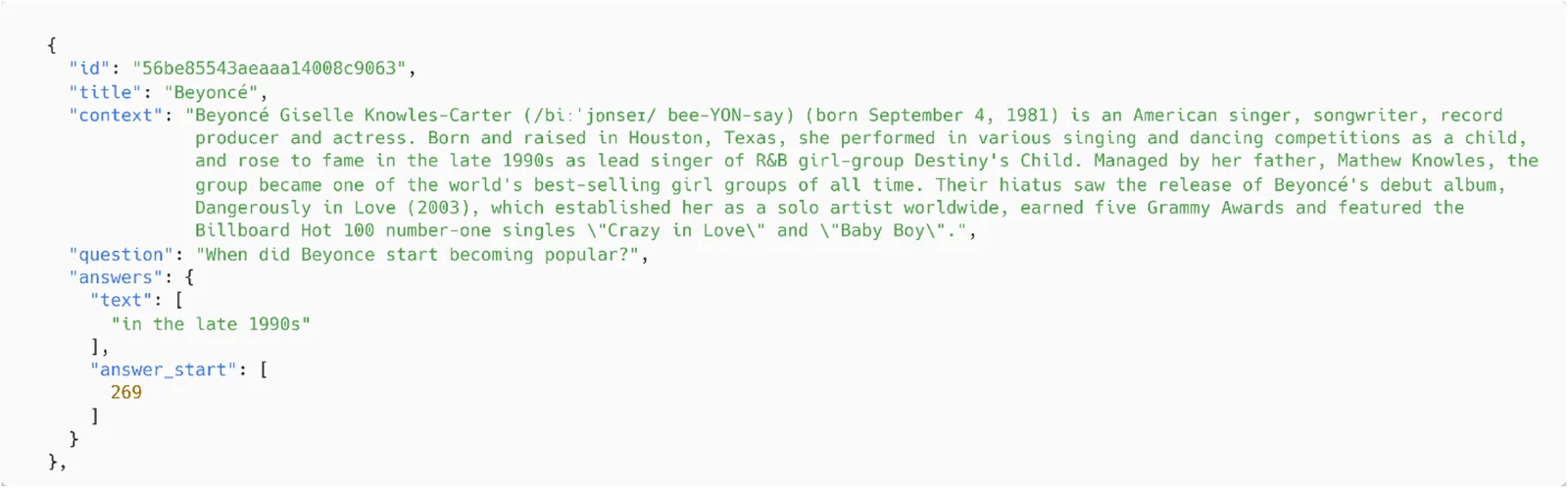
Example content of a full, answerable, record from the SQuAD v2.^15,24^

By comparison, our MagQA data set comprises 168,080 QA pairs and contexts about magnetic materials and their Curie and Néel temperatures. This data set was autogenerated using the methodology presented in this paper. The use of the MagQA data set within the fine-tuning process ensures transfer learning of the BERT models that is specific to the domain of magnetic materials.

Six BERT models were finetuned for QA tasks using three combinations of QA data sets: (i) only SQuAD v2; (ii) only our MagQA data set; (iii) both SQuAD v2 and our MagQA data set. These three combinations were tested against two instances of the BERT-base-cased model: its vanilla form (the BERT-base-cased model from Huggingface) and our DAPT-generated form of this model, to which we refer as the MagBERT-base-cased model. A breakdown of each model and its methods of domain adaptation or otherwise was given in Table 1.

Each BERT model was finetuned for QA tasks using 4 Nvidia A100 GPUs, on a single node until model losses began to show convergence (this required 12–15 epochs); a checkpoint was logged at each epoch. Finetuning each BERT model required between 1 and 3 node hours and the key hyperparameters used for the finetuning process are shown in Table 4. To ensure consistency and to fully explore the difference in domain-adaption techniques, we chose not to differ hyperparameters between models or finetuning data sets.

**Table 4:** Hyperparameters Used in the Finetuning Process for All BERT Models Presented in This Study

Hyperparameter	Value
learning rate	4e-5
max_seq_length	384
per_device_train_batch_size	32
warmup_ratio	0.2
Seed	50

### Autogenerating a Large Domain-Specific Question-Answering Data Set

A range of domain-specific databases of material-property relationships have recently been autogenerated using the ChemDataExtractor toolkit, all of which were publicly released to enable the data-driven design and discovery of novel materials.^25–31^ In subsequent work, algorithms have been created to convert these domain-specific materials databases into QA data sets for finetuning.^14,20^ These algorithms trace back each data record in the materials database to the location in the paper where it was originally reported, and then extract the surrounding sentence as context for the QA data record. The materials data set of Curie and Néel temperatures used for the autogeneration of QA pairs in this study^32^ differs from those previously autogenerated with ChemDataExtractor in one key regard. Namely, the Snowball v2 parser^33,34^ was used for data extraction which, by default, includes the sentence from which the material-property relationship was extracted in the record. The quality of this source database was evaluated in the original study,^32^ which reported a precision of 72% and recall of 61%. Due to our QA-generation algorithm implementing a deterministic template substitution over these records, the precision of the source database presents a lower bound to the correctness of answerable MagQA pairs. This is because only coreference resolution errors present in a source record propagate to its derived QA pairs; meanwhile, no new factual content is introduced. An example record entry of this materials data set is shown in Figure 4.

**Figure 4:**
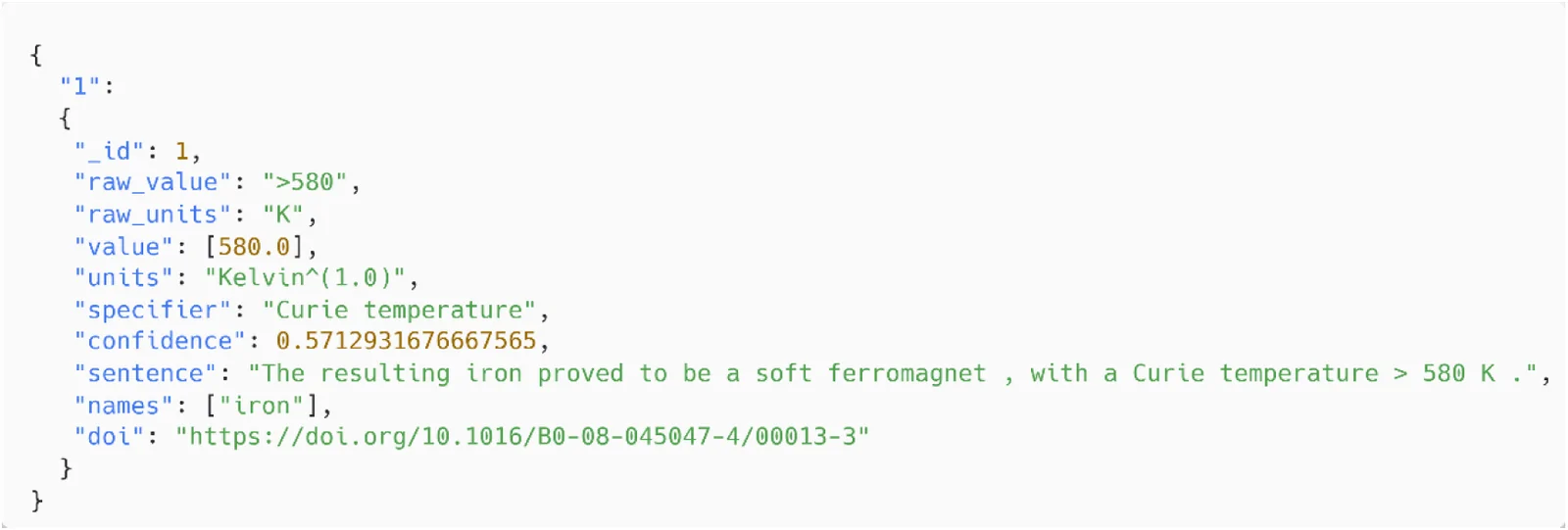
Example of a full data record within our materials database of Curie and Néel temperatures.^32^

This information is converted into a full QA pair data record by first storing the sentence as the context. The algorithm then uses base templates for the first and second-turn questions, and substitutes information where necessary. These templates are as follows:

First-turn QA pair:

- **Question:** What is the <specifier> of <compound name>?
- Answer: <value> <units>

Second-turn QA pair:

- **Question:** What material has a <specifier> of <value> <units>?
- Answer: <compound name>

The specifier refers to the string of text that the parser found, which identified the sentence as a possible candidate for material-property extraction. The use of the specifier as a variable within the QA pair adds variance to the data records. Possible values for the specifier are T_C_, T_N_, T_Curie_, T_Néel_, T_Neel_, Curie temperature, Néel temperature and Neel temperature. With the context and QA pair now determined, the last required part of the record is the start of the answer. This answer is located by using rules based on regular expressions (python regex) to index the sentence context, which reveals the location therein at which the answer begins.

Once all the information has been gathered for a full record, the algorithm formats the data in accordance with the SQuAD v2 format and assigns a unique id and a title of “autogenerated_qa_data” to distinguish the records generated by this algorithm with ones from the SQuAD v2 data set. An example of a first and second-turn QA pair data set record can be seen in Figure 5. Overall, this algorithm yielded 112,043 QA pairs from 56,037 records.

**Figure 5:**
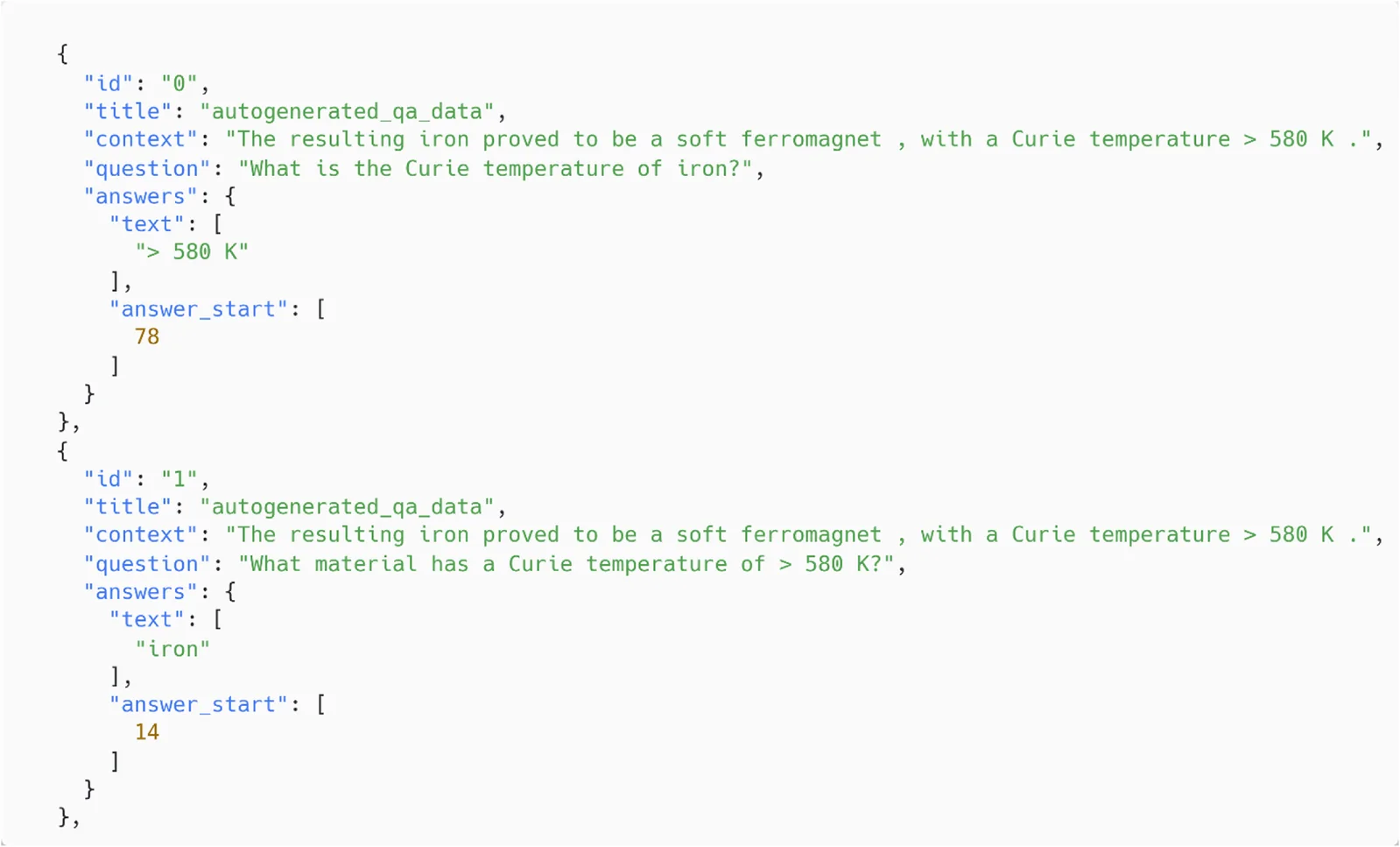
Example extract of the MagQA data set, showing first- and second-turn questions that have been autogenerated from a record of our materials data set about Curie and Néel temperatures.^32^

In accordance with the SQuAD v2 proportioning, our algorithm also generates unanswerable questions in a similarly balanced 2:1 ratio approximately. Thereby, when an unanswerable question is to be generated for a given context, the algorithm randomly selects another record from the database and checks for the following criteria:

- The context of the randomly selected record is not the same as the context for the unanswerable question.
- The answer in the randomly selected record is not present in the unanswerable question context.

If both criteria are met, the algorithm concludes that the question in the randomly selected record cannot be answered by the information given in the context. A new, unanswerable record is then formed with the given context and updated unanswerable question. If neither criteria are met, another random record is selected until such a case where both criteria are met. An example of an unanswerable QA pair is given in Figure 6.

**Figure 6:**
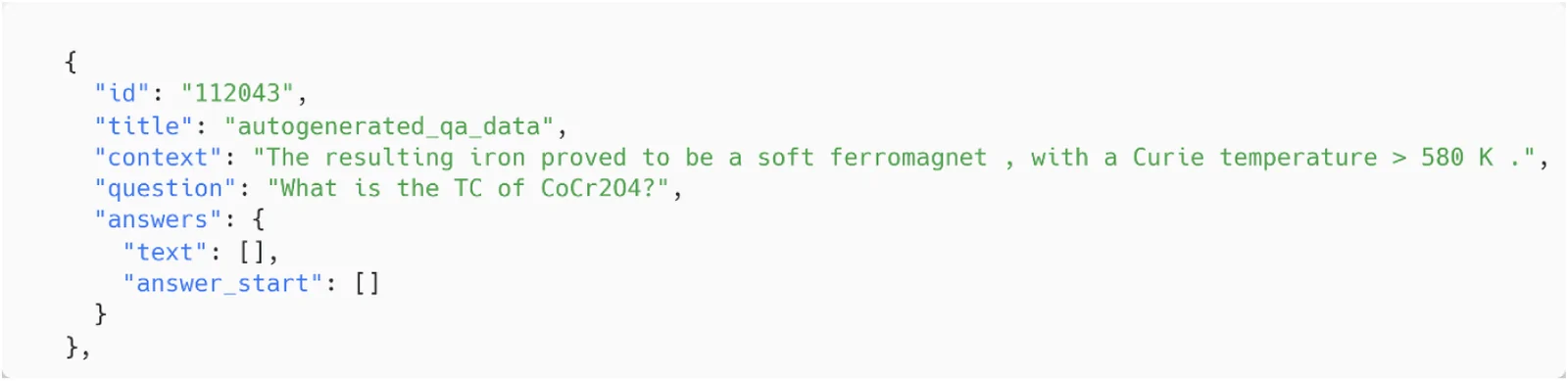
Example record of the MagQA data set, showing an autogenerated unanswerable question.

The algorithm generated 112,043 answerable records of first and second-turn questions, and 56,037 unanswerable records, giving a total domain-specific data set size of 168,080 records. The 130,319 QA pairs in SQuAD v2 train split were then added into this data set to create a *mixed data set* that was shuffled to ensure randomness between MagQA and SQuAD v2 QA pairs throughout the fine-tuning process; this mixed data set contained 298,399 records. It should be noted here that the domain-specific contexts in the autogenerated MagQA data set differ from those contained within SQuAD v2. The contexts in our data set span only single sentences. In contrast, the contexts given in SQuAD v2 span multiple sentences, with even the possibility of the answer and question being in separate sentences. However, the larger quantity of domain-specific questions are expected to be sufficient for combating this disadvantage.

### Finetuning Larger BERT Architectures

The herein presented methodology of finetuning BERT models for QA tasks details how to autogenerate domain-specific BERT models for the domain of magnetic materials. While we have discussed the reasoning behind choosing BERT as our model architecture, it is important to show how our work can be generalized to larger architectures. As such, we opted to finetune vanilla RoBERTa-base and DeBERTa-base model architectures on the same QA data set that had yielded our highest performing MagBERT QA model with our methodology. Table 5 presents a comparison of the key parameters involved in the different BERT model architectures that were employed.

**Table 5:** Key Parameters of BERT-Base, RoBerta-Base and DeBerta-Base Model Architectures Used in This Study

Parameter	BERT-base	RoBERTa-base	DeBERTa-base
Number of hidden layers	12	12	12
Hidden size	768	768	768
Number of attention heads	12	12	12
Total number of parameters	∼110 million	∼125 million	∼139 million

### Ablation Study to Understand QA Data Set Size Requirements

Our custom algorithms autogenerated a data set of 168,080 QA pairs; this number is directly dependent on the number of data records that reside in the materials database from which the QA data set is created. In a real-world scenario, the size of a QA data set can vary greatly and thus we finetuned our BERT models on ablations of our QA data set: with 10,000, 50,000 and 100,000 QA pairs alongside the fully sized data set of 168,080 QA pairs. In common with the extrapolation of our work to larger BERT model architectures, we applied this ablation study to our MagBERT model that performs best on QA tasks. The QA data sets for the ablation study were obtained by iteratively reducing the full MagQA data set from the finetuning stage to its first 100,000, 50,000 and then 10,000 QA pairs. Each of the resulting three QA data sets were then randomly mixed with the SQuAD v2 train split, and three ablation BERT models were finetuned according to the same procedure outlined earlier in the Fine-Tuning for QA Tasks section.

### Evaluation Metrics and Method

#### Manually Curating a Small Domain-Specific QA Validation Data Set for Technical Evaluation

The relative performance of all six BERT models, once finetuned for QA tasks, needs to be evaluated. This requires a domain-specific QA validation data set. As stated earlier, a QA data set requires four entries for every record: a context, a question, an answer, and the start of the answer within the context.

The contexts for our validation QA data set were obtained by randomly selecting a subset of the Royal Society of Chemistry papers which are contained within the parent corpus about magnetic materials that is employed in this work. By construction, we note that all 150 of these evaluation papers are therefore contained within the corpus used for DAPT, meaning that the three DAPT models were exposed to the evaluation contexts during MLM pretraining. Any resulting bias favors the DAPT models; notably these models nonetheless underperform their non-DAPT counterparts (*cf.* Results and Discussion), adding strength to our argument that finetuning is the more impactful stage of domain adaptation.

In accordance with data entries in SQuAD v2, the contexts in each record in our validation QA data set spanned multiple sentences and contained at least one material-property relationship that consists of a Curie or Néel temperature. Each context was consolidated into a Comma Separated Value (CSV) file, which was uploaded to QA Annotator,^3,35^ a tool written for the purpose of annotating contexts for QA data sets.

For each material-property relationship present in each context, a first-turn and second-turn QA pair were generated. The first-turn QA pair has been formatted such that the question contains the material, and the answer contains the property; in this case, a Curie or Néel temperature. This order is reversed for the second-turn QA pair, with the question containing the temperature, and the answer containing the material; this order reversal, together with the conditional formulation of the second-turn QA on the first-turn QA (see introduction), enables the determination of material and property relationships. Example usage of QA Annotator is given in Figure 7, which displays a context taken from a paper by Shi et al.,^36^ together with its generated first-turn and second-turn QA pairs. From the 150 contexts that were used, a total of 439 answerable QA pairs were created.

**Figure 7:**
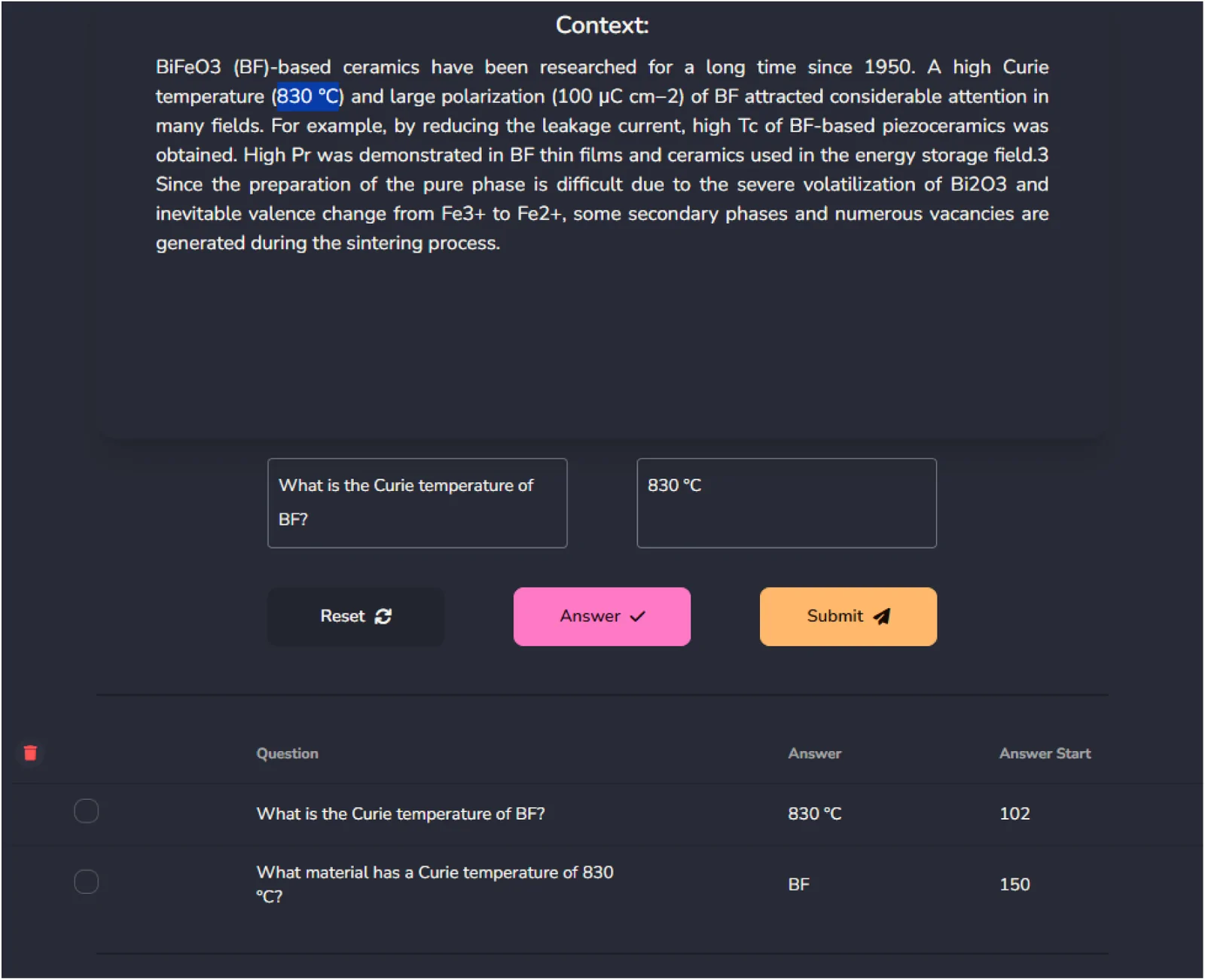
Example usage of QA Annotator.^3,35^ A context^36^ is given in the context box. A first-turn question is written in the question box. The answer to the first-turn question is highlighted in the context. Both the first-turn and second-turn QA pairs are given at the bottom, with the first-turn answer starting at location 102, and the second-turn answer starting at location 150.

QA pairs are generated using QA Annotator, by manually typing question into the question box, and the answer to the question is highlighted with the mouse cursor. Clicking the *Answer* button saves the answer and finally clicking the *Submit* button adds the QA pair, along with the start of the answer to the QA pairing list. Once this process had been completed for every context in the uploaded CSV file for this study, all the contexts, QA pairs, and answer starts were downloaded from the tool into a CSV file. A Python script was then used to convert the CSV entries into a format that followed that of SQuAD v2 and exported into a JSON file to produce the manually curated validation data set. An example extract from the data set is provided in Figure 8.

**Figure 8:**
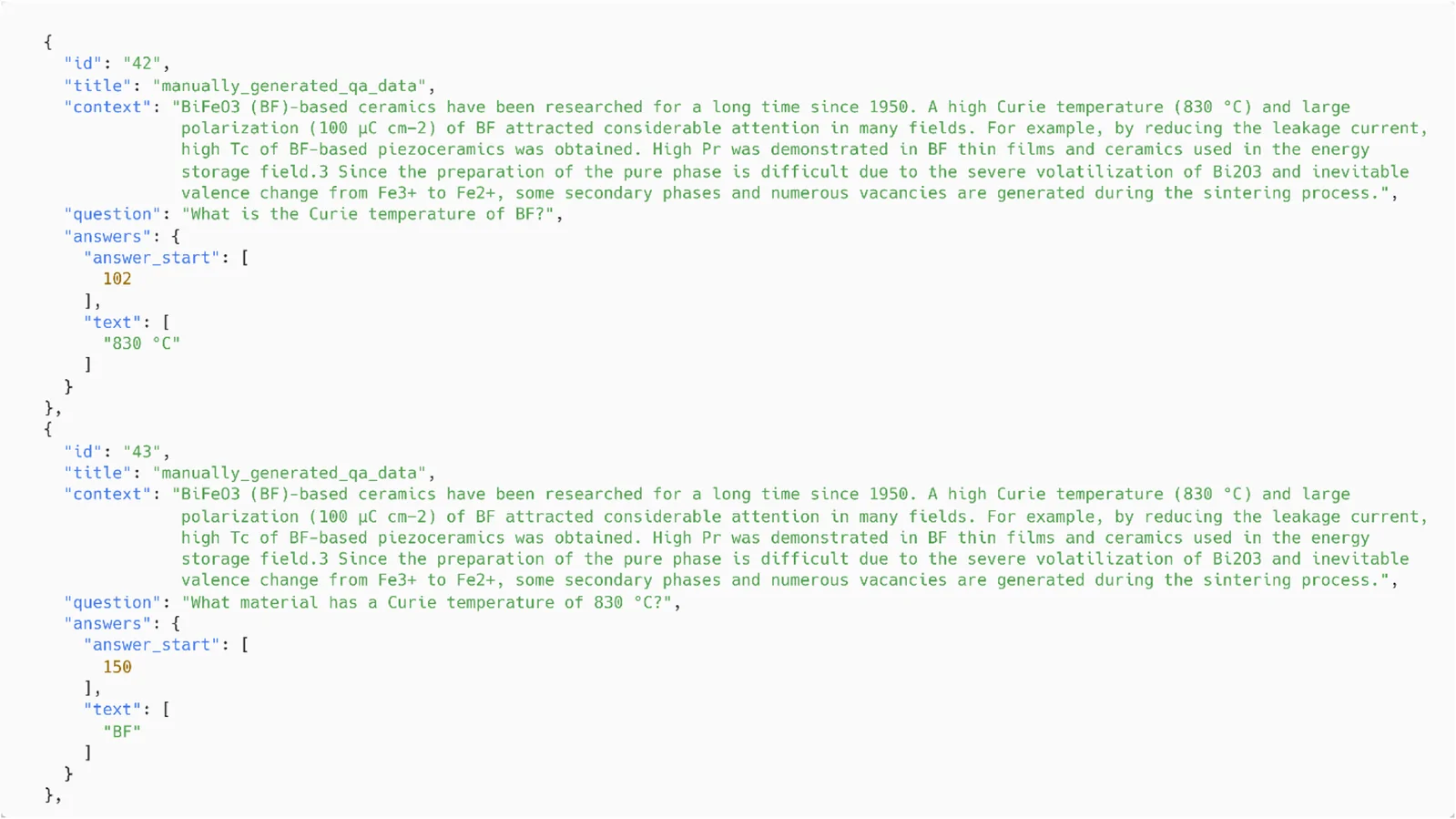
Example of a first-turn and second-turn record of our manually labeled evaluation data set, showing the context,^36^ a first-turn QA pair and answer start, and a second-turn QA pair and answer start.

Once the 439 answerable questions were created with QA Annotator, unanswerable questions were created using the same algorithm that was used to create MagQA described in the Autogenerating a Large Domain-Specific Question-Answering Data Set section. As with MagQA, the unanswerable questions were created at a 2:1 ratio to match the distribution of SQuAD v2. This resulted in a manually annotated evaluation data set comprising 659 QA pairs, of which 439 were answerable, and 220 were unanswerable. It should be noted here that we have adopted a bipartite design in this study: our autogenerated MagQA data set, whose contexts span single sentences, was used strictly for finetuning, while this held-out, manually curated data set, whose contexts span multiple sentences, was reserved exclusively for model evaluation; at no point was MagQA split into a train and test portions. This design was chosen deliberately, both to demonstrate that LLMs finetuned for materials-science QA on autogenerated QA data sets with single-sentence contexts can achieve high performance when presented with multisentence QA tasks, and to determine how many epochs of finetuning on the autogenerated QA pairs are required to produce a converged model.

All BERT models were fine-tuned for QA tasks using the QA data sets described in Table 1, until the losses began to show convergence, which occurred between 12 and 15 epochs; this ensured that the best possible performance was achieved for each model. A checkpoint was taken at each epoch, and each checkpoint was evaluated on the manually curated validation set. The checkpoint with the best *F*1 score for each model was chosen as the final checkpoint used in this study. The best performing checkpoint for each BERT model is outlined in Table 6.

**Table 6:** The 6 Language Models Presented in This Study, the Epoch Affording the Best Performing Version of the BERT Model That was Used for This Study, and the Number of Node Hours That Had Surpassed When That Epoch was Reached

Model	Epoch	Finetuning node hours for the epoch affording the best performing BERT model
BERT_SQuADv2	10	3
MagBERT_DAPT_SQuADv2	11	3
MagBERT_DAPT_MagQA_Mixed	1	1
MagBERT_DAPT_MagQA	1	1
MagBERT_MagQA	1	1
MagBERT_MagQA_Mixed	1	1

This *F*1 score is one of two standard metrics that are used for our technical evaluation; the other is an exact-match score. The *F*1 score is defined as a harmonic mean of precision and recall at the token level, describing the quality of the predicted answer against the ground-truth answer. An exact-match score is quantified as the percentage of predicted answers that exactly match the ground-truth answer stated in the data set record. We further extended our evaluation by providing these metrics for both answerable and unanswerable questions for each model, alongside the overall scores.

## Results and Discussion

### Technical Validation and Testing

Figures 9 and 10 show the *F*1 and exact-match scores for all 6 BERT models when evaluated on the manually curated QA validation data set. The MagBERT_MagQA_Mixed model performed best, *i.e*., the BERT model fine-tuned on both our autogenerated MagQA data set and SQuAD v2, with no DAPT; this model achieved an *F*1 score of 78.43% and an exact-match score of 72.84%. In contrast, the BERT_SQuADv2 model performed worst of all 6 models. This is as expected since the finetuning and pretraining stage of this model had been focused on generic English language (SQuAD v2), *i.e*., it contained no domain-specific data.

**Figure 9:**
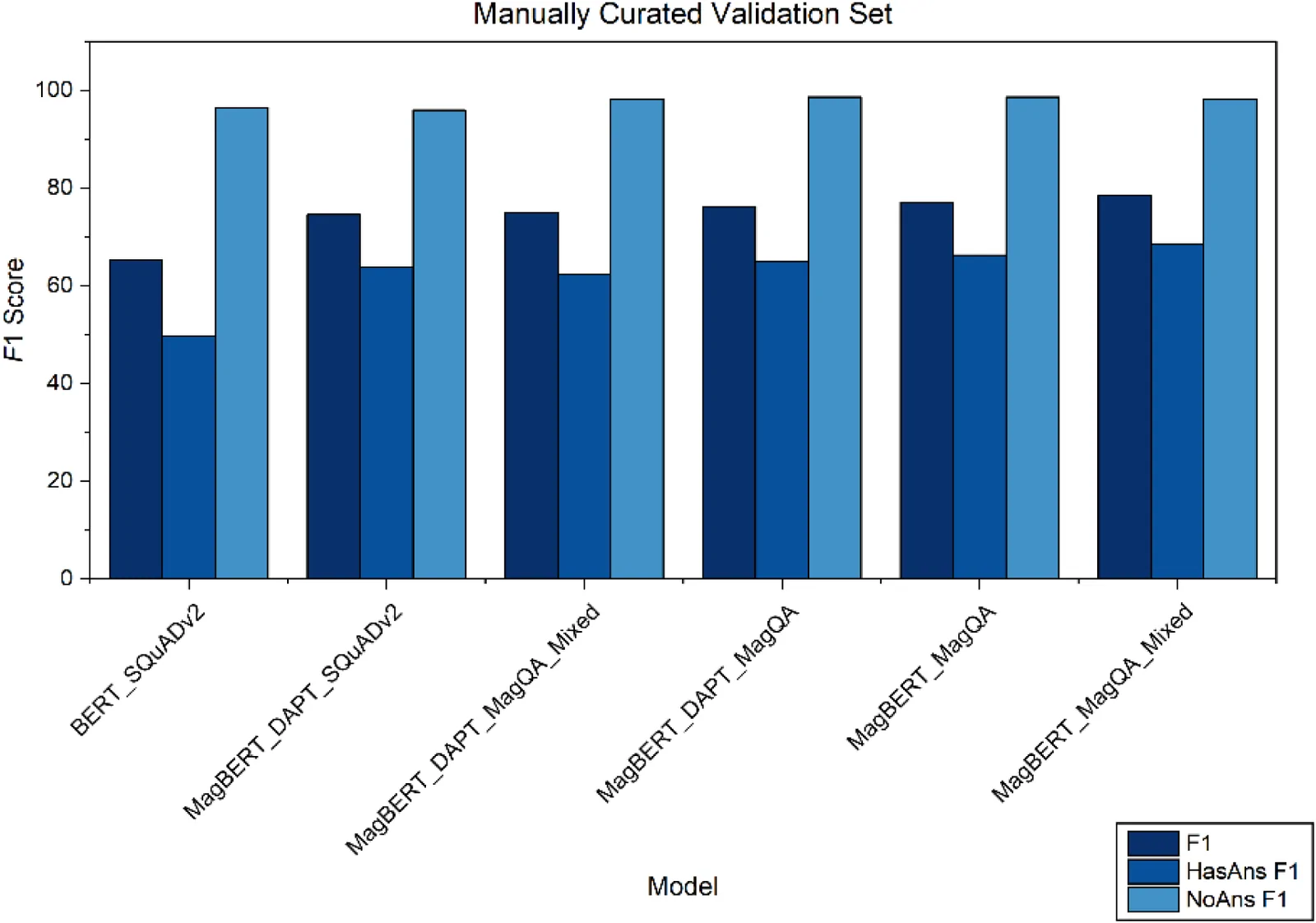
*F*1 scores for the 6 BERT models presented in this study when evaluated on the manually curated validation data set. Metrics for questions with answers are labeled “HasAns” and metrics for questions with no answers are labeled “NoAns”.

**Figure 10:**
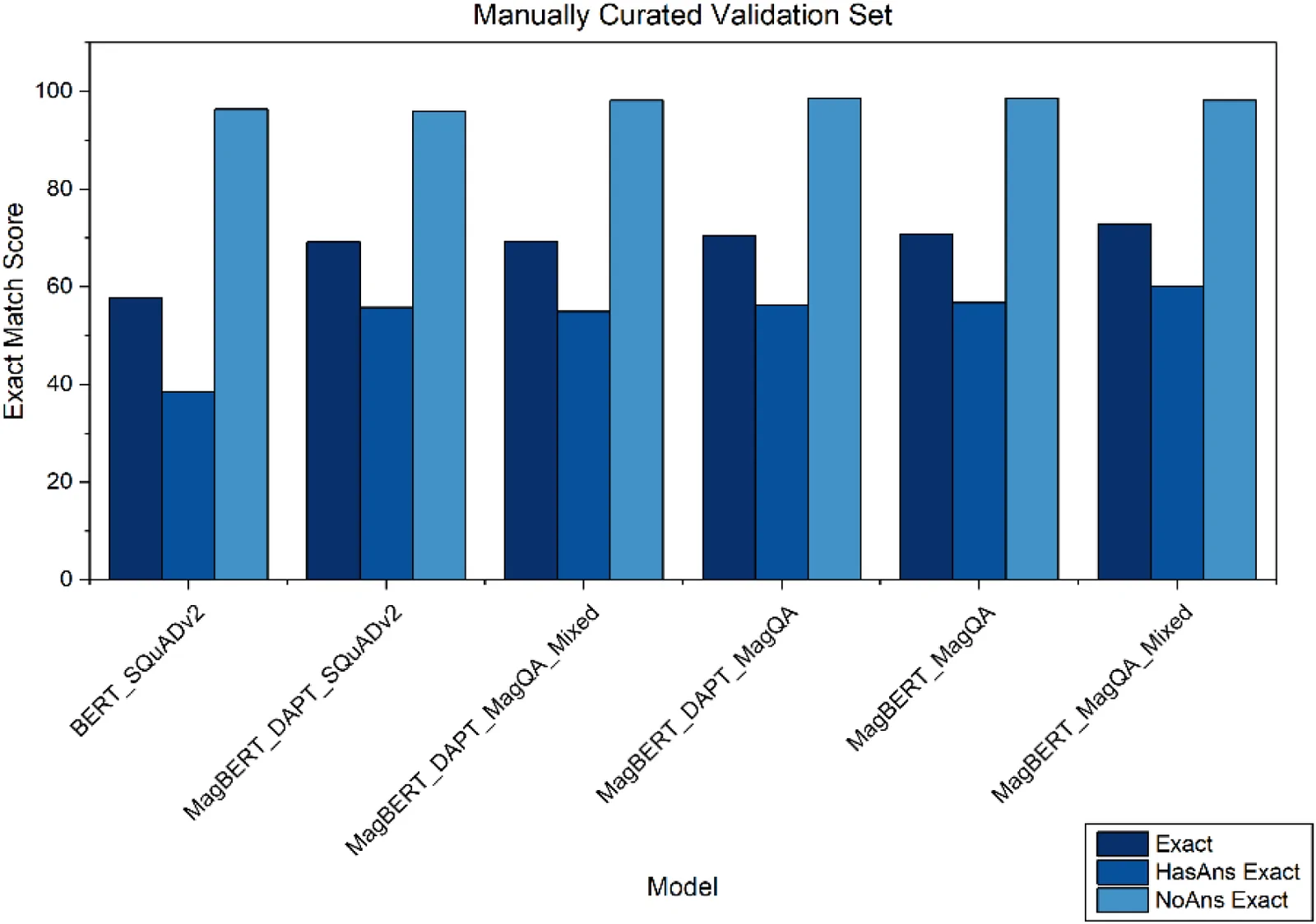
Exact-match scores for the 6 BERT models presented in this study when evaluated on the manually curated validation data set. Metrics for questions with answers are labeled “HasAns” and metrics for questions with no answers are labeled “NoAns”.

Models that have been domain-adapted in some way, whether via a pretraining or finetuning process, or via both processes, all exhibited *F*1 scores >74% and exact-match scores >69%, compared to our BERT_SQuADv2 model (65.32% and 57.81%, respectively) that had not been domain adapted via either process. This shows that language models benefit significantly from added domain-specific knowledge when finetuned for the downstream task of QA.

The inclusion of domain-specific knowledge solely in the pretraining (DAPT) stage showed a significant improvement on models that were only finetuned on generic English language in the form of SQuADv2, *cf*. the MagBERT_DAPT_SQuADv2 model achieves *F*1 and exact-match scores of 74.55% and 69.20%, respectively, compared with the performance of the BERT_SQuADv2 model (65.32% and 57.81%, respectively). However, this type of performance improvement did not carry over when the BERT models included domain-specific information in both the DAPT and finetuning stages of optimization. For example, the MagBERT_DAPT_MagQA_Mixed and MagBERT_DAPT_MagQA models, which were both domain-adapted pretrained, and finetuned on the MagQA data set, achieved *F*1 scores of 74.94% and 76.25% respectively, and exact-match scores of 69.35% and 70.41%, respectively, on the manually curated QA validation data set. These scores are only marginally lower than those of their non-DAPT counterparts, *cf*. the MagBERT_MagQA model scores an *F*1 and exact-match score of 77.09% and 70.71%, and the MagBERT_MagQA_Mixed scores an *F*1 score of 78.43% and an exact-match score of 72.84%. This indicates that, when a model is being domain-adapted through finetuning, domain-specific data in the pretraining stage (i.e., DAPT) can be counter-productive. The higher evaluation scores for the MagBERT_MagQA_Mixed model, which was finetuned on both our MagQA data set and SQuAD v2, compared to those of the MagBERT_MagQA model, which was only finetuned on our MagQA data set, shows that the addition of generic English language QA pairs into the finetuning stage of BERT model optimization increases performance in answering domain-specific questions.

It should be noted that all BERT models with some form of domain adaptation achieved competitive performance relative to one another, with differences between highest and lowest performing domain-adapted models being <4% in both *F*1 and exact-match scores. This trend continues across scoring metrics for both questions that have answers and those that do not; this indicates a consistent adaptation of weights in the neural networks within the BERT models presented in this study. Considering only the domain-adapted BERT models, the MagBERT_DAPT_SQuADv2 model, which was not finetuned on our MagQA data set, achieved the lowest scores for both *F*1 and exact-match metrics, while the MagBERT_MagQA_Mixed model achieved the highest scores. This demonstrates that a much greater impact is realized if one imparts domain-specific data within the finetuning stage than the pretraining stage of BERT model optimization; and adding additional generic English QA paired knowledge can further increase BERT model performance.

Since the reported scores correspond to the epoch with the highest *F*1 score on the manually curated evaluation data set (*cf.* Table 6), it is important to quantify the extent to which this checkpoint selection influences the reported comparison. Table 7 therefore presents, for each BERT model, the best epoch scores alongside the scores at the final trained epoch and the mean ± standard deviation across all trained epochs. The decline in performance from the best to the final epoch (4.3–7.8% *F*1 score) is observed for all 6 models, including those finetuned solely on SQuAD v2, and is consistent with overfitting upon extended finetuning. Importantly, the ordering of the models by their mean *F*1 score across all epochs reproduces the ordering of their best epoch scores, with only the two DAPT MagQA models swapping places. Another observation is that the BERT_SQuADv2 model ranks last at every epoch. This indicates that checkpoint selection does not drive the comparative conclusions of this study. While it must be noted that the differences in *F*1 score among the domain-adapted models are comparable to their variation across epochs, our ranking of these models is consistent with prior studies.^14,20^ We compare our work to these studies in more detail in the discussion.

**Table 7:** 6 Language Models Presented in This Study, and Their *F*1 and Exact-Match Scores at the Best-Performing and Final Epochs, as Well as the Mean and Standard Deviation Across All Epochs, When Evaluated on the Manually Curated Evaluation Dataset

Model	Best epoch	Best epoch *F*1(%)	Best epoch EM (%)	Final epoch	Final epoch *F*1(%)	Final epoch EM (%)	Mean *F*1 ± s.d. (%)	Mean EM ± s.d. (%)
BERT_SQuADv2	10	65.32	57.81	15	61.02	53.72	61.58 ± 2.98	54.81 ± 1.99
MagBERT_DAPT_SQuADv2	11	74.55	69.20	15	69.52	64.18	70.51 ± 3.72	65.24 ± 3.60
MagBERT_DAPT_MagQA_Mixed	1	74.94	69.35	12	70.36	67.07	71.55 ± 1.33	68.21 ± 0.98
MagBERT_DAPT_MagQA	1	76.25	70.41	12	69.19	65.40	71.06 ± 2.60	67.13 ± 2.25
MagBERT_MagQA	1	77.09	70.71	11	72.41	68.74	72.77 ± 3.09	68.69 ± 2.60
MagBERT_MagQA_Mixed	1	78.43	72.84	12	70.67	68.13	73.02 ± 3.48	69.64 ± 2.87

### Technical Validation of BERT Models Evaluated on SQuAD V2

Figures 11 and 12 show the *F*1 and exact-match scores for all 6 BERT models when evaluated on the validation split of the SQuAD v2 data set. As expected, the BERT_SQuADv2 model, with no domain-specific information imparted into the pretraining or finetuning stages, achieved the best performance against this general English QA validation data set, giving *F*1 and exact-match scores of 71.75% and 67.84%, respectively. Once DAPT was added to the process to afford the MagBERT_DAPT_SQuADv2 model, very little change was observed, with *F*1 and exact-match scores of 71.55% and 67.48% being observed.

**Figure 11:**
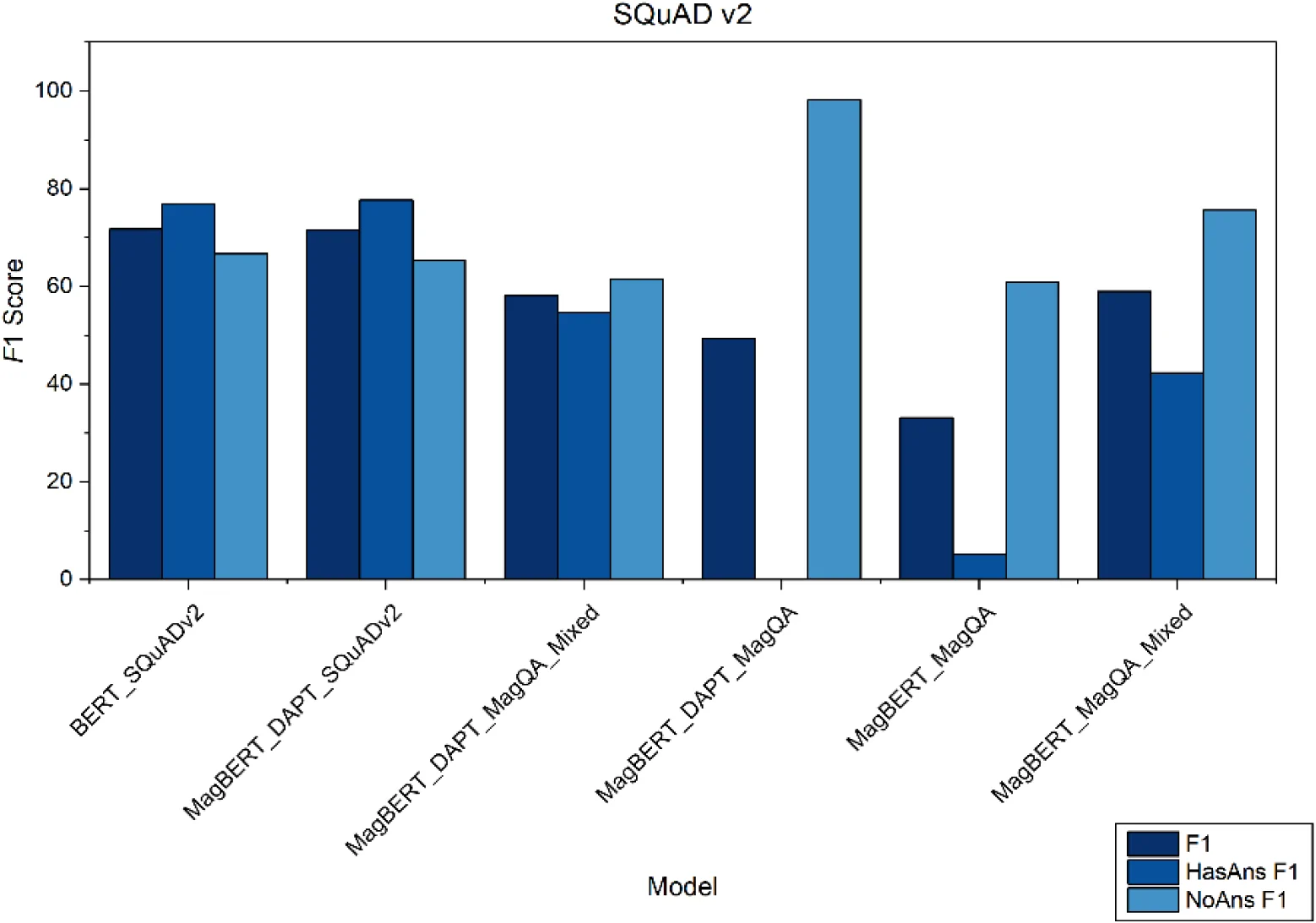
*F*1 scores for the 6 BERT models presented in this study when evaluated against the SQuAD v2 benchmark. Metrics for questions with answers are labeled “HasAns” and metrics for questions with no answers are labeled “NoAns”.

**Figure 12:**
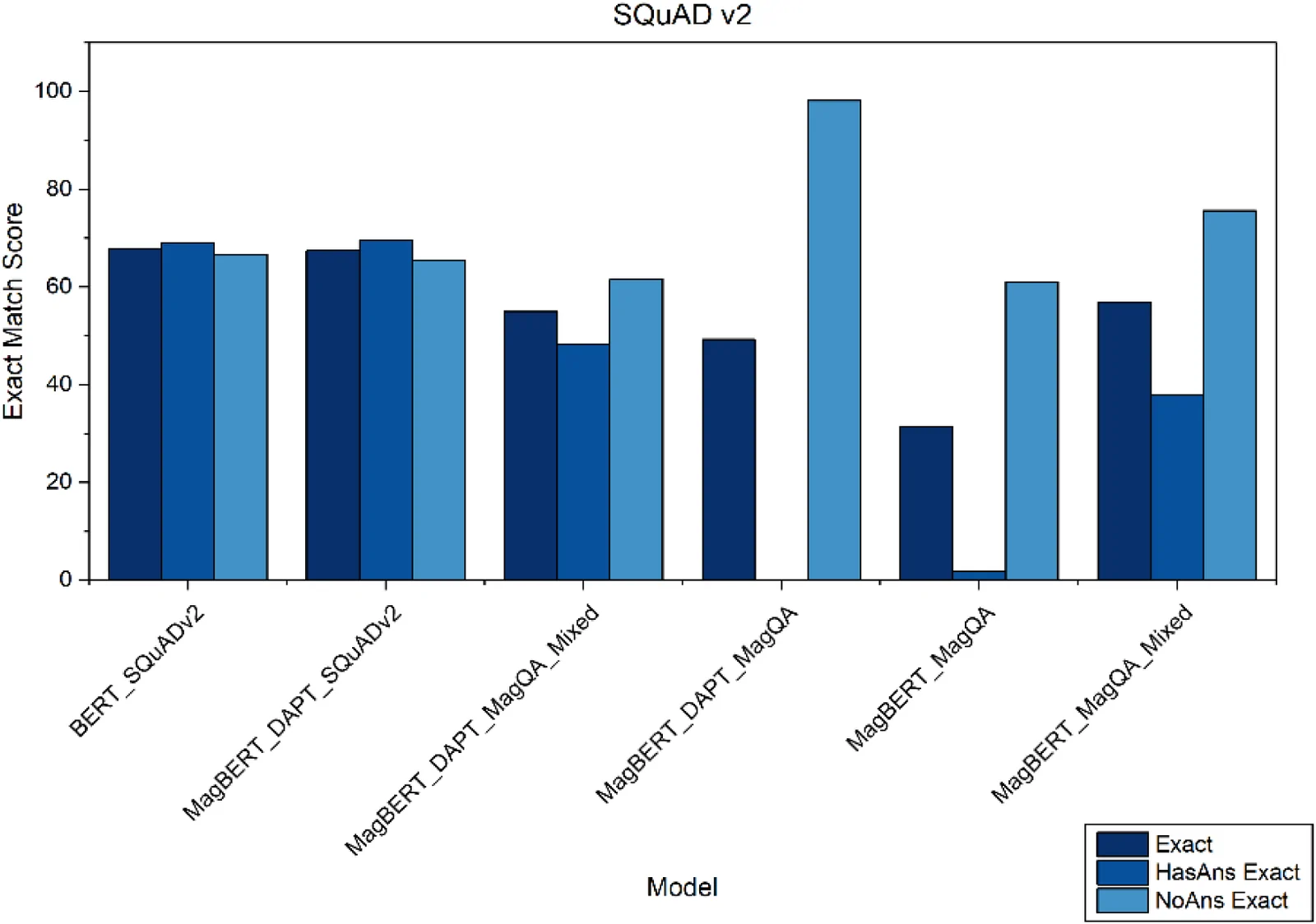
Exact-match scores for the 6 BERT models presented in this study when evaluated against the SQuAD v2 benchmark. Metrics for questions with answers are labeled “HasAns” and metrics for questions with no answers are labeled “NoAns”.

Once domain-specific information was imparted into the finetuning stage of model optimization, a considerable drop in performance was observed, relative to the scores of BERT models that had only been finetuned on SQuAD v2. For example, the MagBERT_DAPT_MagQA_Mixed model achieved *F*1 and exact-match scores of 58.12% and 54.97%, respectively, and its non-DAPT counterpart, the MagBERT_MagQA_Mixed model, achieved marginally higher *F*1 and exact-match scores of 58.99% and 56.80%; all of these scores are ∼14% lower than those of models that had only been finetuned against SQuAD v2 for downstream QA tasks. These results further evidence that finetuning is more impactful than pretraining when creating domain-specific BERT models, and that finetuning should include domain-specific data for optimal results.

As expected, all BERT models that had not been finetuned on generic English language performed poorly when evaluated on the SQuAD v2 benchmark. DAPT showed performance improvement, as demonstrated by a comparison of the MagBERT_DAPT_MagQA model, with *F*1 and exact-match scores of 49.33% and 49.25% over its non-DAPT counterpart, the MagBERT_MagQA model, which achieved *F*1 and exact-match scores of 33.10% and 31.40%, respectively. However, both of these BERT models performed very poorly with questions that have answers, to the extent that the MagBERT_DAPT_MagQA model failed to answer a single answerable question correctly. The MagBERT_MagQA model performed marginally better with answerable questions, but considerably worse with unanswerable questions relative to its DAPT counterpart. The difference in context types between the MagQA and SQuAD v2 data sets should be reiterated here. The contexts contained in SQuAD v2 span multiple sentences of generic English text in the form of paragraphs, in contrast to our MagQA data set where contexts span only a single, domain-specific sentence.

Our finding that the MagBERT_DAPT_MagQA model failed to answer any answerable question correctly was initially a surprising result. We sought to understand why this happened, by comparing the attention mappings of our BERT_SQuADv2 and MagBERT_DAPT_MagQA models. Thereby, attention maps are a visualization of how strongly different tokens attend to one another, with brighter colors showing stronger attention, and darker colors showing weaker attention. The specific attention mappings that we provide in Figures 13 and 14 consist of the average attention across the final 3 layers of these two BERT models; these layers were selected as they tend to be the most task-specific with earlier layers typically having broader attention. These attention mappings show 3 answerable records from the SQuAD v2 validation split, that our BERT_SQuADv2 model was able to answer correctly, but MagBERT_DAPT_MagQA was not. The answer tokens to each question within the context tokens are highlighted in green and the 3 questions themselves are shown as plots a), b) and c) on the *y*-axes of Figures 13 and 14.

**Figure 13:**
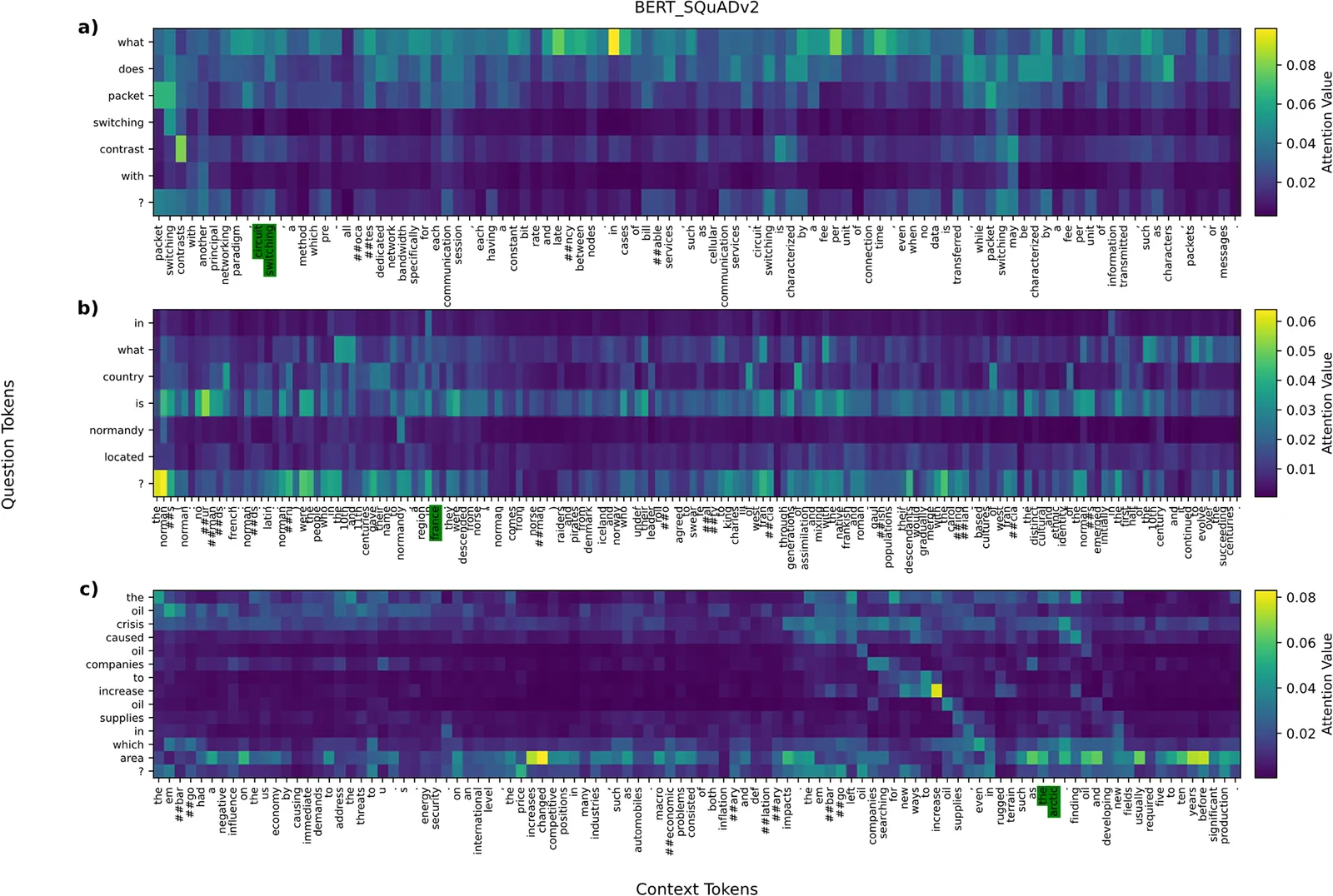
Attention mappings of the last 3 layers of the BERT_SQuADv2 model architecture presented in this study for the three context-question pairs a, b and c. The answer span within the context is highlighted in green.

**Figure 14:**
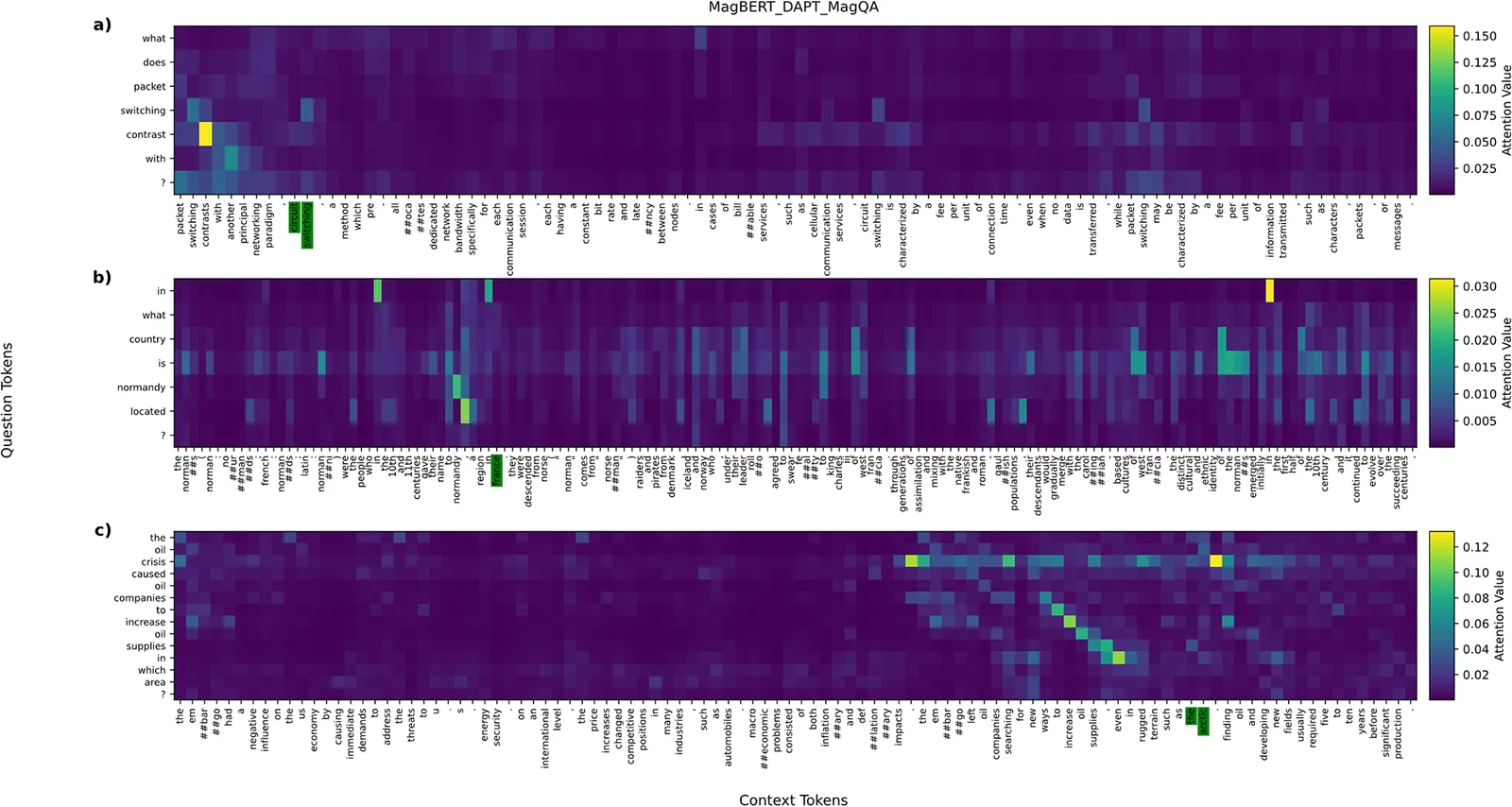
Attention mappings of the last 3 layers of the MagBERT_DAPT_MagQA model architecture presented in this study for the three context-question pairs a, b and c. The answer span within the context is highlighted in green.

Figures 13 and Figure 14 show a stark contrast in the level of attention between the tokens in the question and the tokens in the context for the 3 extracts of SQuADv2. For example, Figure 13a shows that the model was able to answer correctly the question, “What does packet switching contrast with?”. Thereby, strong attention can be seen between the answer tokens in the context. “circuit” and “switching”, and tokens in the question such as “what” and “does”. This shows that the BERT_SQuADv2 model has adapted its weights sufficiently to understand the semantic relationship between the tokens in which it is presented. Compare this to Figure 14a, where very weak attention can be seen between the same tokens spanning the answer in the context and the same important tokens in the question: this indicates that model weights were not sufficiently adapted for the downstream task of generic English language QA.

While Figure 14 shows significantly weaker attention around the answer tokens for the MagBERT_DAPT_MagQA model than Figure 13 shows for the BERT_SQuADv2 model, the figures also show a general trend across the attention for all tokens in all 3 context-question pairs. It can be seen that the overall attention for all tokens in Figure 14 is significantly weaker than Figure 13 for all 3 context-question pairs that we tested. Consequently, we can clearly deduce that the QA pairs in the MagQA data set are insufficient to adapt the weights for the downstream task of generic English language QA. This explains the importance of concatenating SQuAD v2 and our MagQA data set to provide an increase in performance of our domain-specific BERT models on QA tasks. This finding is in line with similar studies performed on BERT models within other materials-science domains.^14,20^

### Technical Validation of RoBERTa-Base and DeBERTa-Base Models

So far, this work has showcased the performance of the vanilla BERT-base model architecture once finetuned for domain-specific material-property extraction in the field of magnetism. However, it is important to test our workflow on other BERT model architectures to see how well our methodology translates. To this end, we finetuned the larger, and newer, RoBERTa-base and DeBERTa-base model architectures on our highest performing QA data set (MagQA_mixed) for magnetism, to create the MagRoBERTa_MagQA_Mixed and MagDeBERTa_MagQA_Mixed models. The RoBERTa and DeBERTa architectures from the BERT family naturally contain some similarities. Key similarities and differences are listed in Table 5. In particular, the RoBERTa and DeBERTa architectures are larger versions of BERT, containing more parameters within their respective model. Our evaluation procedure for these larger finetuned models followed that of the aforementioned family of MagBERT models, i.e., a checkpoint was taken at each epoch of the finetuning stage, and the epoch with the highest *F*1 score on the manually curated validation set was selected as the final model. Table 8 shows each language model and the epoch at which it delivered its highest performance, along with comparable results from our MagBERT_MagQA_Mixed and BERT_SQuADv2 models.

**Table 8:** Four Language Models Presented in the Technical Validation of RoBERTa and DeBERTa Architectures, the Epoch That Afforded the Best Performing Model for This Study, and the Number of Node Hours That Had Surpassed When That Epoch was Reached

Model	Epoch	Finetuning node hours for the epoch affording the best performing language model
BERT_SQuADv2	10	3
MagBERT_MagQA_Mixed	1	1
MagRoBERTa_ MagQA_Mixed	1	1
MagDeBERTa_ MagQA_Mixed	2	2

Figures 15 and Figure 16 respectively show the *F*1 scores and exact-match scores for our domain-specific RoBERTa and DeBERTa models when evaluated on our manually curated validation data set. Our highest performing MagBERT model is also included as a comparative reference.

**Figure 15:**
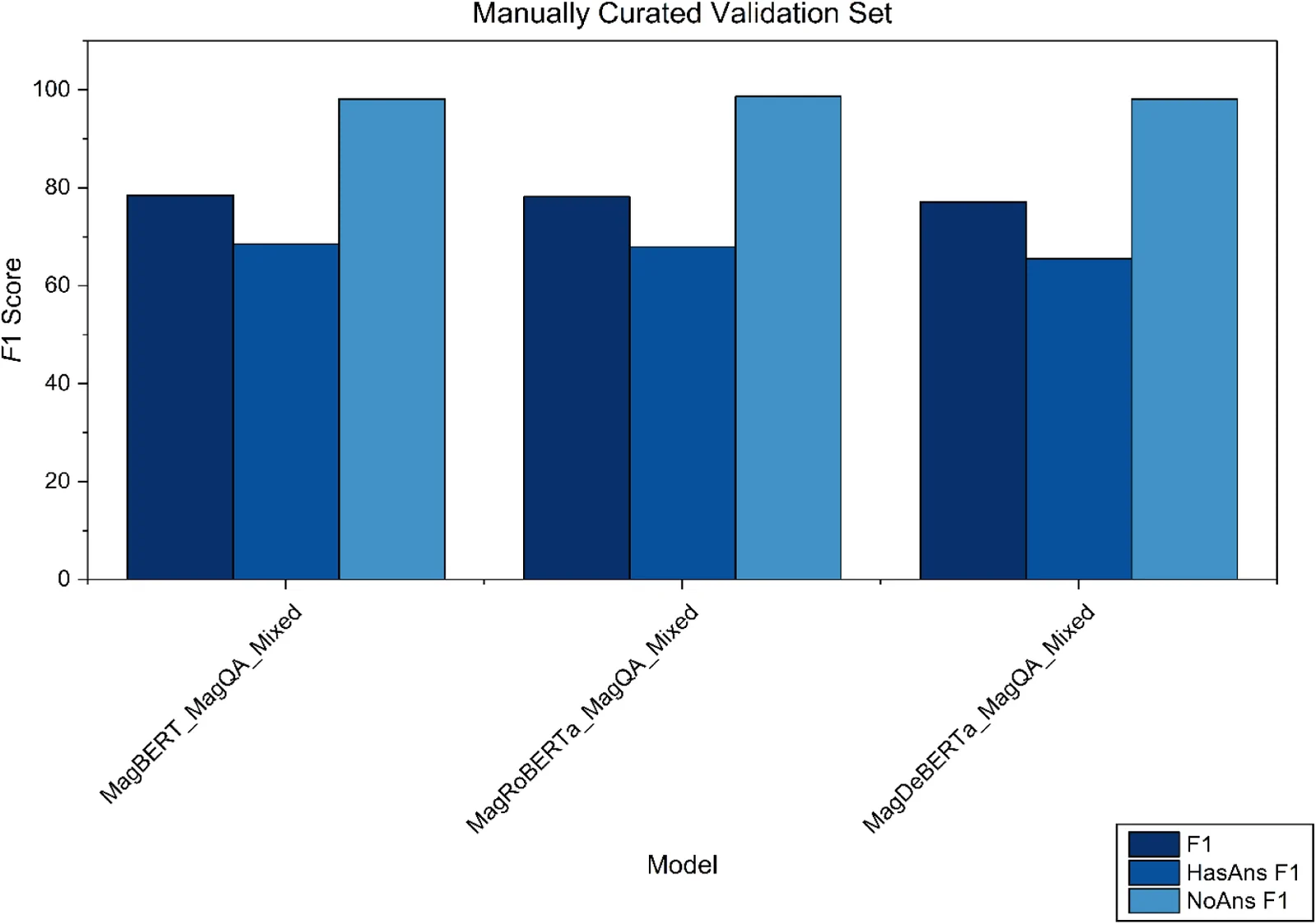
F1 scores for the MagBERT_MagQA_Mixed, MagRoBERTa_MagQA_Mixed and MagDeBERTa_MagQA_Mixed models presented in this study when evaluated on the manually curated validation data set. Metrics for questions with answers are labeled “HasAns” and metrics for questions with no answers are labeled “NoAns”.

**Figure 16:**
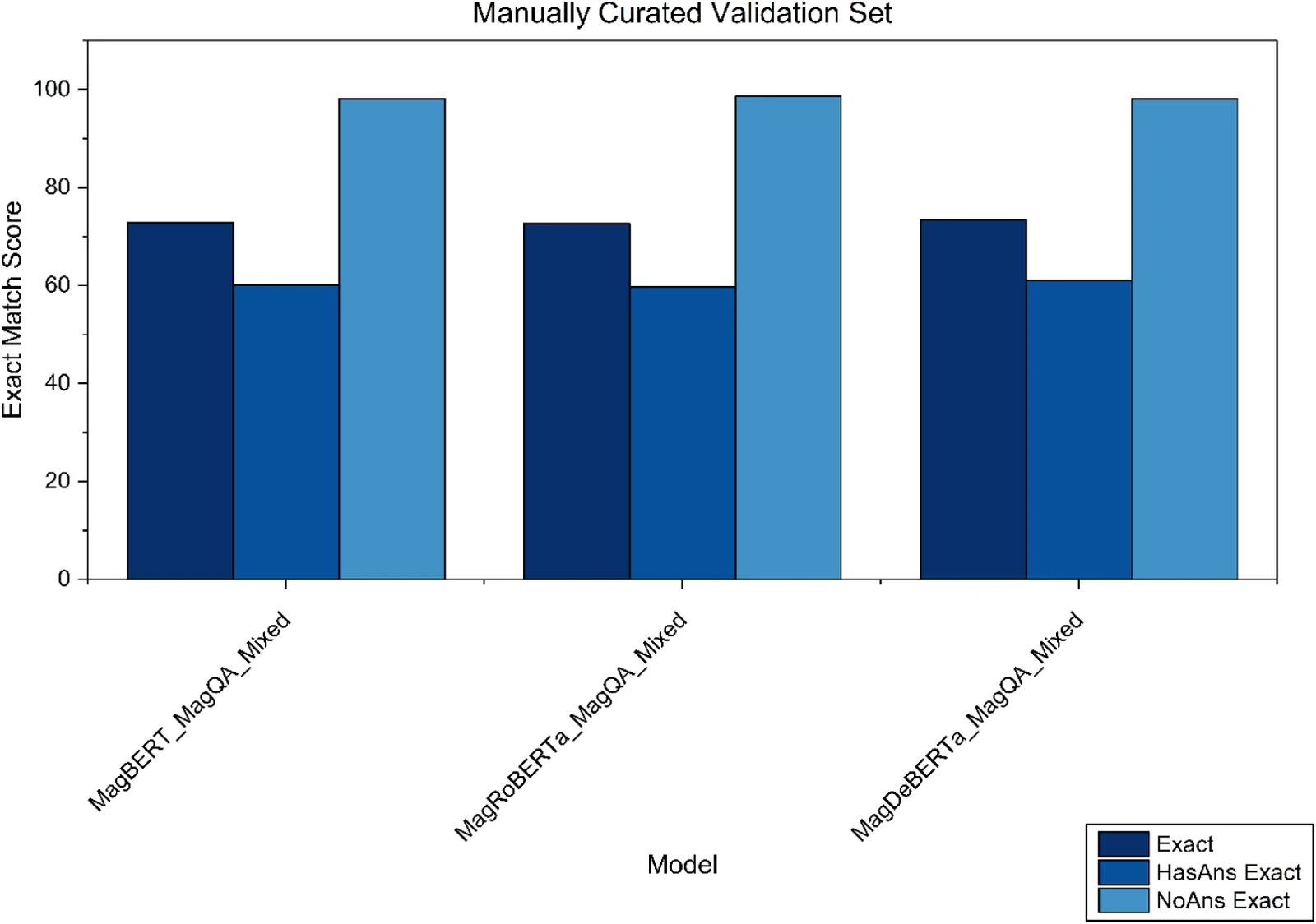
Exact-match scores for the MagBERT_MagQA_Mixed, MagRoBERTa_MagQA_Mixed, MagDeBERTa_MagQA_Mixed models presented in this study when evaluated on the manually curated validation data set. Metrics for questions with answers are labeled “HasAns” and metrics for questions with no answers are labeled “NoAns”.

Consistent performance was achieved for all three models across the board, with MagRoBERTa_MagQA_Mixed and MagDeBERTa_MagQA_Mixed models exhibiting *F*1 scores of 78.16% and 77.12% and exact-match scores of 72.69% and 73.44%, respectively. For comparison, the MagBERT_MagQA_Mixed model achieved a similar *F*1 score and exact-match score of 78.43% and 72.84%, respectively.

As with Table 7, Table 9 reports the best epoch scores of the MagRoBERTa_MagQA_Mixed and MagDeBERTa_MagQA_Mixed models alongside their final epoch scores as well as their means and standard deviations across all trained epochs, with the MagBERT_MagQA_Mixed model included as a comparative reference. These larger architectures exhibit the same behavior that was observed for the BERT_base models: performance peaks within the first two epochs and declines thereafter (best-to-final-epoch differences of 6.9% and 6.0% in *F*1 score, respectively), consistent with overfitting upon extended finetuning, while the mean *F*1 scores of all three models fall within 0.2% of one another. This corroborates our findings that the performance of domain-specific BERT models on domain-specific QA tasks is not influenced by the size of the BERT model architecture, and demonstrates that this conclusion is not an artifact induced by checkpoint selection.

**Table 9:** Three Language Models Presented in the Technical Validation of RoBERTa and DeBERTa Architectures and Their *F*1 and Exact-Match Scores at the Best-Performing and Final Epochs, as Well as the Mean and Standard Deviation Across All Epochs, When Evaluated on the Manually Curated Evaluation Dataset

Model	Best epoch	Best epoch *F*1(%)	Best epoch EM (%)	Final epoch	Final epoch *F*1(%)	Final-epoch EM (%)	Mean *F*1 ± s.d. (%)	Mean EM ± s.d. (%)
MagBERT_MagQA_Mixed	1	78.43	72.84	12	70.67	68.13	73.02 ± 3.48	69.64 ± 2.87
MagRoBERTa_MagQA_Mixed	1	78.16	72.69	12	71.27	68.13	72.87 ± 2.13	69.35 ± 1.49
MagDeBERTa_MagQA_Mixed	2	77.12	73.44	12	71.17	68.44	72.85 ± 2.65	69.73 ± 2.31

Figures 17 and 18 show the *F*1 scores and exact-match scores for our domain-specific RoBERTa and DeBERTa models when evaluated on the validation split of the SQuADv2 data set. Our highest performing MagBERT model, as well as our vanilla BERT model finetuned on SQuAD v2, have also been included as a comparative reference. Thus, the performance of the domain-specific BERT models on domain-specific QA tasks does not appear to be influenced by the size of the BERT model architecture.

**Figure 17:**
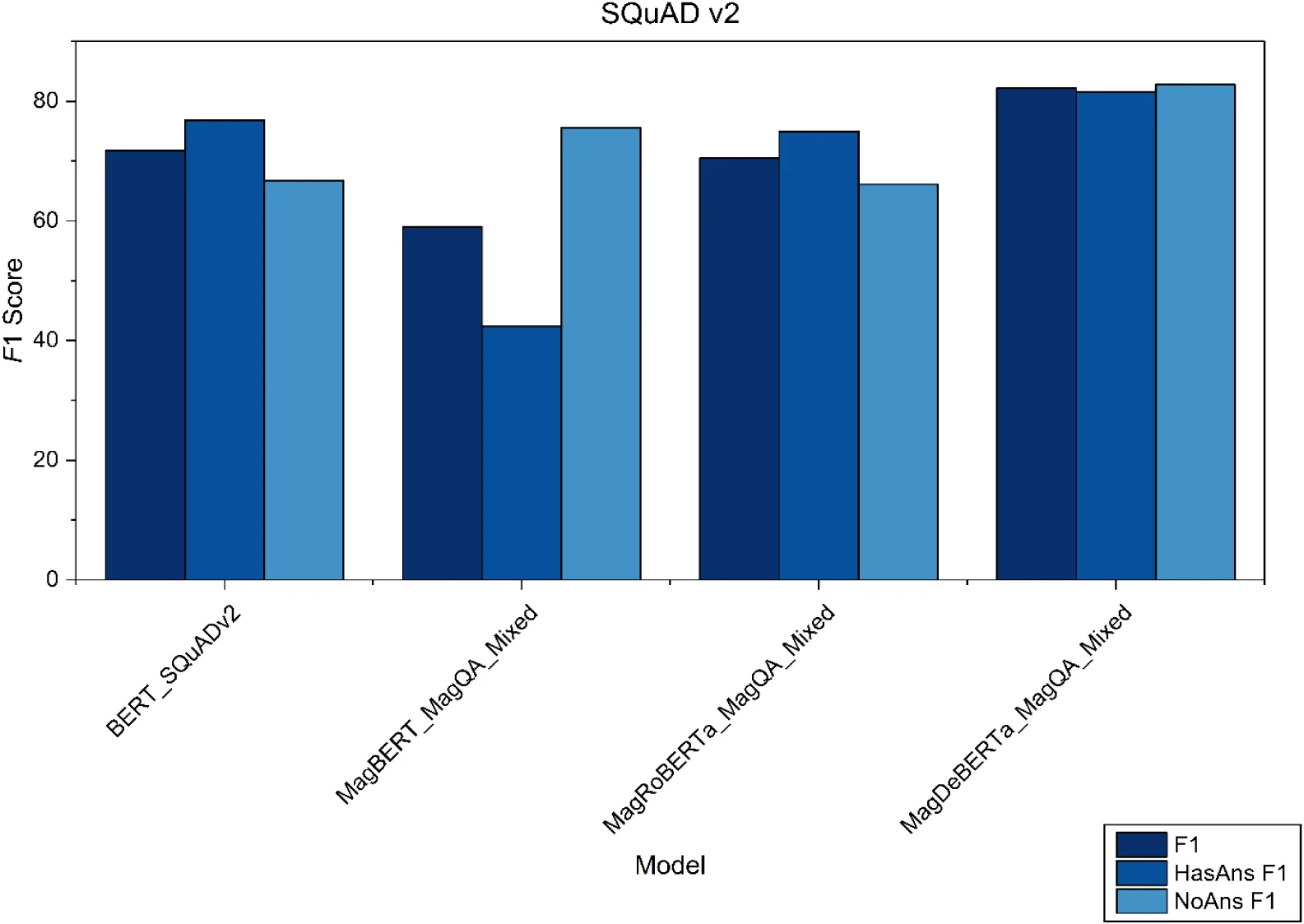
F1 scores for the BERT_SQuADv2, MagBERT_MagQA_Mixed, MagRoBERTa_MagQA_Mixed and MagDeBERTa_MagQA_Mixed models presented in this study when evaluated against the SQuAD v2 benchmark. Metrics for questions with answers are labeled “HasAns” and metrics for questions with no answers are labeled “NoAns”.

**Figure 18:**
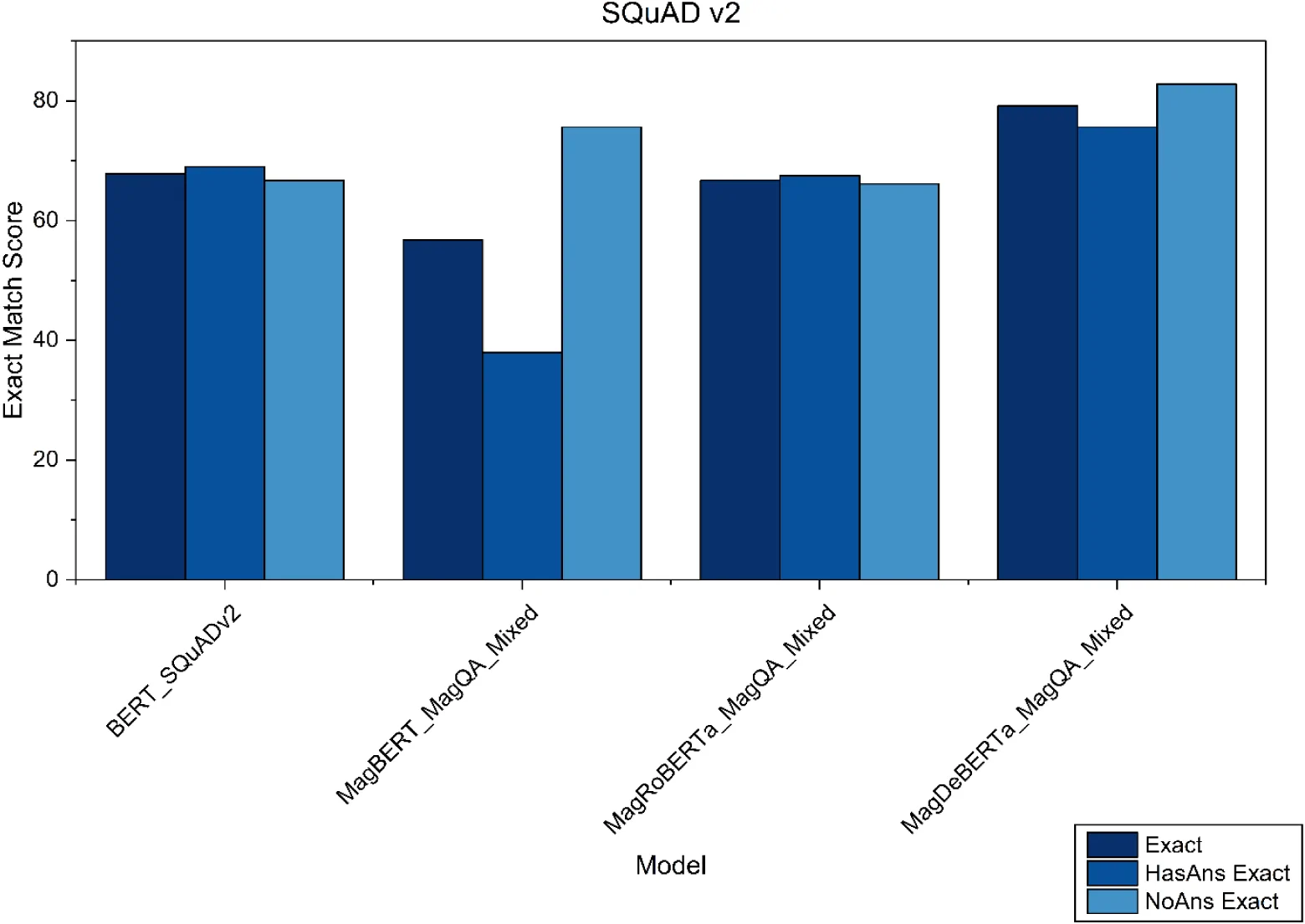
Exact-match scores for the MagRoBERTa_MagQA_Mixed, MagBERT_MagQA_Mixed and DeBERTa_MagQA_Mixed models presented in this study when evaluated against the SQuAD v2 benchmark. Metrics for questions with answers are labeled “HasAns” and metrics for questions with no answers are labeled “NoAns”.

By stark contrast, there are clear differences in the behavior of the models of different sizes when they were evaluated on the nondomain-specific SQuAD v2. Thereby, both our MagRoBERTa and MagDeBERTa models significantly outperform our highest performing MagBERT model when tested on generic English language QA pairs; with the MagRoBERTa_MagQA_Mixed and MagDeBERTa_MagQA_Mixed models achieving *F*1 scores of 70.49% and 82.19%, and exact-match scores of 66.78% and 79.2%, respectively, while our MagBERT_MagQA_Mixed model achieved *F*1 and exact-match scores of 58.99% and 56.80%, respectively. Interestingly, our MagRoBERTa_MagQA_Mixed model achieved very similar performance to our BERT_SQuADv2 model (*F*1 score of 71.75%, exact-match score of 67.84%), despite having domain-specific QA pairs included in the finetuning training data – an observation that is not present in the scores of the MagBERT_MagQA_Mixed model. Thus, the performance of domain-specific BERT models on *non*-domain-specific QA tasks *is* significantly influenced by the size of the BERT model architecture. We revisit this observation in more detail in the Discussion section.

### Technical Validation of an Ablation Study to Understand QA Data Set Size Requirements

Our MagQA data set presented herein consists of 168,080 domain-specific QA pairs. This number stems directly from the quantity of records contained within the magnetic domain-specific materials database from which they were autogenerated. This predetermined quantity of QA pairs raises the logistical question of how many QA pairs are needed for the performance of domain-specific material-property extraction with domain-specific BERT models to be unaffected? To this end, we conducted an ablation study, whereby the first 10,000, 50,000 and 100,000 QA pairs in the MagQA data set were selected and used to finetune the vanilla BERT-base model via our methodology presented in this study. Each ablated QA data set was augmented with the train split of SQuAD v2 and randomly mixed prior to the finetuning process.

Figures 19 and Figure 20 show the *F*1 scores and exact-match scores for the three ablated models, together with the cognate metrics from our highest performing MagBERT model that had been finetuned on the full MagQA data set, *i.e*., the MagBERT_MagQA_Mixed model, which serves as a comparative reference.

**Figure 19:**
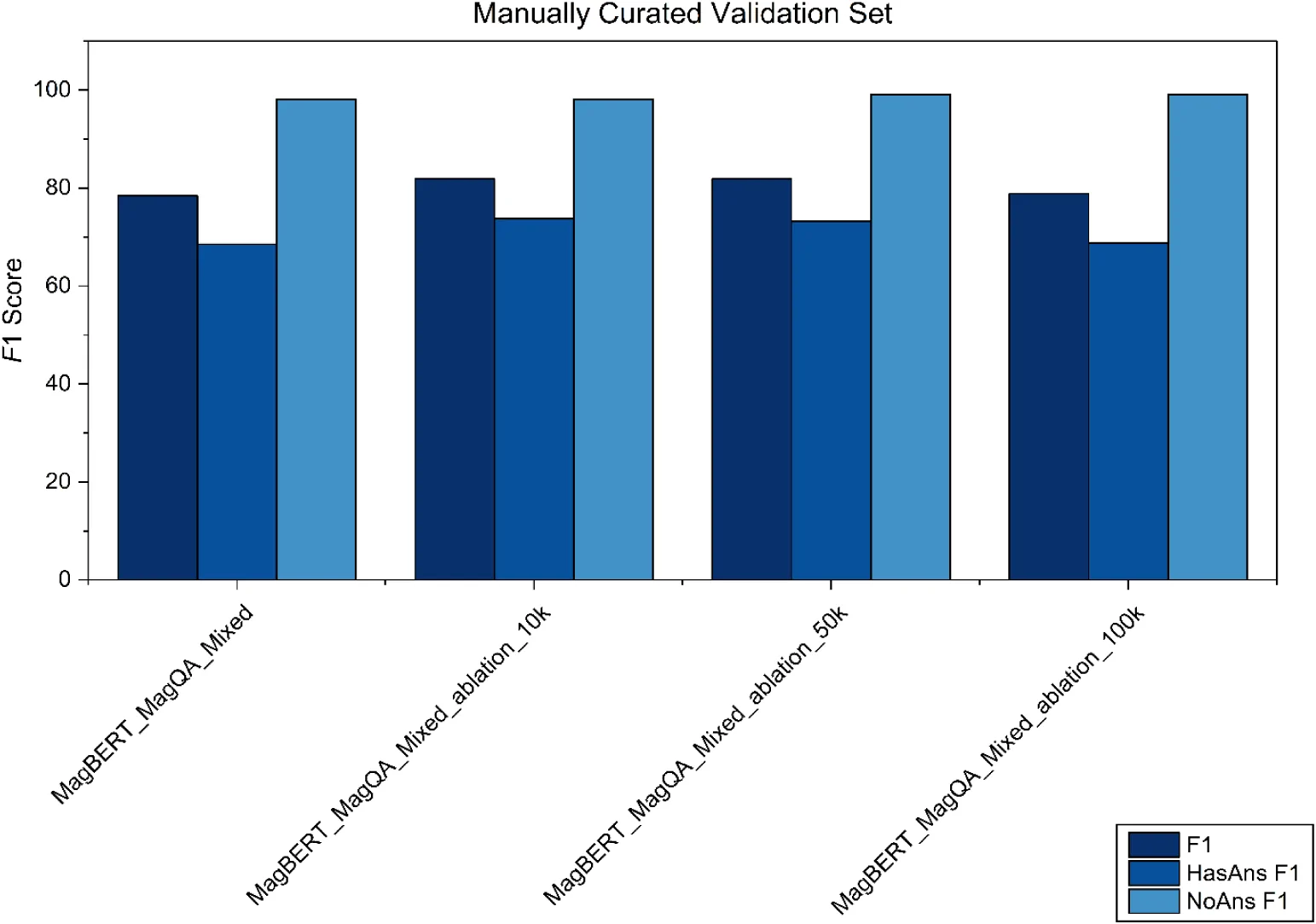
*F*1 scores for the MagBERT_MagQA_Mixed model, finetuned on the full MagQA data set, together with the cognate results from the three ablation models explored in this study. Metrics for questions with answers are labeled “HasAns” and metrics for questions with no answers are labeled “NoAns”.

**Figure 20:**
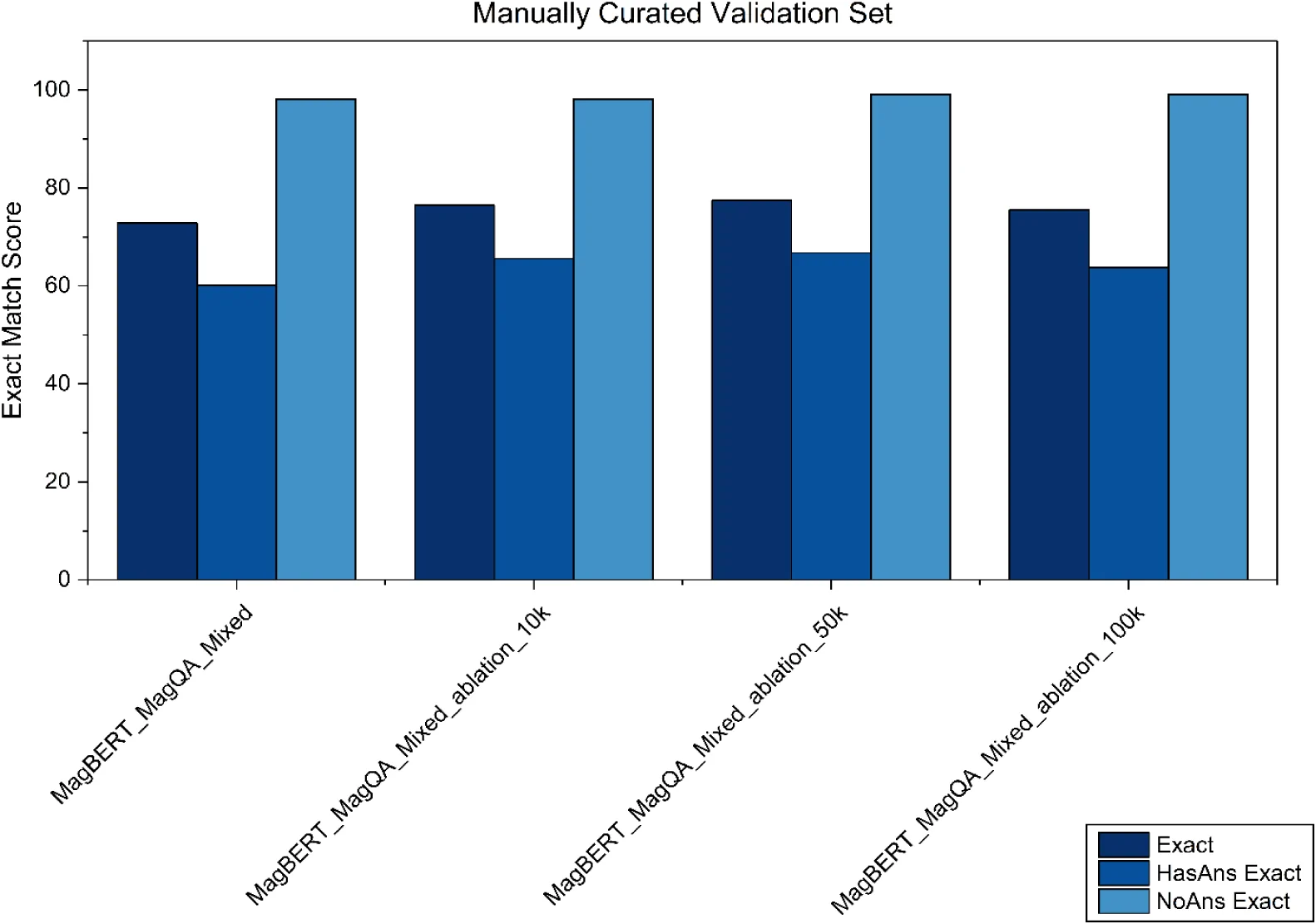
Exact-match scores for the MagBERT_MagQA_Mixed model, finetuned on the full MagQA data set, together with the cognate results from the three ablation models explored in this study. Metrics for questions with answers are labeled “HasAns” and metrics for questions with no answers are labeled “NoAns”.

The ablation models were all subject to the same evaluation procedure used throughout this paper, whereby each model was trained for 12 epochs, and the epoch with the best *F*1 score on the manually curated validation data set is the model presented. Table 10 shows each model, the epoch that corresponds to its best performance, and the node hours required for the finetuning process.

**Table 10:** Four Language Models Presented in the Ablation Study^a^

Model	Epoch	Finetuning node hours for the epoch affording the best performing BERT model
MagBERT_MagQA_Mixed	1	1
MagBERT_MagQA_Mixed_ablation_10k	3	1
MagBERT_ MagQA_Mixed_ablation_50k	3	1
MagBERT_ MagQA_ Mixed _ablation_100k	4	2

a The epoch that delivered the best performing model, and the number of node hours that had surpassed when that epoch was reached.

Table 11 further reports the best epoch scores for the three ablation models alongside their final epoch scores, as well as their means and standard deviations. The best-to-final epoch differences (6.2–9.7% in *F*1 score) again evidence the overfitting upon extended finetuning. Notably, the models finetuned on the ablated QA data sets retain their advantage over the model finetuned on the full MagQA data set in their mean scores as well as their best epoch scores, confirming that this observation is not an artifact of checkpoint selection. The comparatively large standard deviation of the model finetuned on the 10,000 QA-pair data set (5.7% *F*1 score) reflects its low score in the first epoch, in accordance with the greater number of epochs that the ablated models required to reach their best performance.

**Table 11:** Four Language Models Presented in the Ablation Study and Their *F*1 and Exact-Match Scores at the Best-Performing and Final Epochs, as Well as the Mean and Standard Deviation Across All Epochs, When Evaluated on the Manually Curated Evaluation Dataset

Model	Best epoch	Best-epoch *F*1(%)	Best-epoch EM (%)	Final epoch	Final epoch *F*1(%)	Final epoch EM (%)	Mean *F*1 ± s.d. (%)	Mean EM ± s.d. (%)
MagBERT_MagQA_Mixed	1	78.43	72.84	12	70.67	68.13	73.02 ± 3.48	69.64 ± 2.87
MagBERT_MagQA_Mixed_ablation_10k	3	81.90	76.48	12	75.71	72.23	77.17 ± 5.74	72.63 ± 6.04
MagBERT_MagQA_Mixed_ablation_50k	3	81.86	77.54	12	75.18	71.02	75.30 ± 3.52	71.38 ± 2.96
MagBERT_MagQA_Mixed_ablation_100k	4	78.90	75.57	12	69.19	66.78	75.04 ± 3.46	71.32 ± 2.87

As one would expect, the ablated models needed more epochs to finetune them than the full data set did: as fewer training data are provided, the model may require more passes of the QA data set to learn the semantic patterns and adjust the weights accordingly. Interestingly, all BERT models achieved comparable performance, with use of the ablated QA data sets outperforming the model which employed the full QA data set, by as much as 3.5% in *F*1 score: whereby, the models finetuned on the 10,000, 50,000 and 100,000 QA data sets achieved scores of 81.9%, 81.86% and 78.9%, respectively, compared to the 78.43% that was achieved by using the full MagQA data set to yield the MagBERT_MagQA_Mixed model. Similar behavior was observed in that of the exact-match scores, with BERT models finetuned using the 10,000, 50,000 and 100,000 QA data sets achieving 76.48%, 77.54% and 75.57%, respectively, compared to the 72.84% achieved by finetuning on the full MagQA model to afford the MagBERT_MagQA_Mixed model. While these differences in statistical figures-of-merit are modest, they are consistent. Moreover, it was not expected that finetuning a BERT model using fewer domain-specific QA data would achieve a higher performing BERT model compared to the case where the same model was tested on the manually curated QA data set. Thus, while the optimal number of QA pairs for finetuning domain-specific BERT models is not the focus of this study, we have attempted to explain these findings in the Discussion section.

## Discussion

This study has investigated the viability and efficacy of using large, autogenerated domain-specific QA data sets and DAPT for transfer learning to create high-performance domain-specific BERT models finetuned for the downstream task of QA. The domain of interest for this specific study is magnetic materials science, although we note that related work on other domains of materials science demonstrates that our findings have more general implications.^14,20^

When evaluated on our manually curated QA validation data set, all BERT models with some form of domain-adaption showed improved performance over that of the BERT_SQuADv2 model, which was not subjected to any domain-adaption. This shows that the weights in the neural network within the BERT models can adapt to better suit scientific literature in the magnetic materials domain. The MagBERT_DAPT_SQuADv2 model possesses domain-specific knowledge which had been imparted solely in the pretraining stage and was only finetuned on generic English text and showed a significant rise in performance over its non-DAPT counterpart, the BERT_SQuADv2 model, proving that DAPT is a viable method for adapting language models to the domain of magnetic materials. However, all BERT models that employed domain-specific data in their finetuning stage performed better than the MagBERT_DAPT_SQuADv2 model. This demonstrates that the weights in the neural network within a BERT model respond more to the finetuning stage than they do to the pretraining process, and that for domain adaption, finetuning is the more important process. This trend carried over when domain-specific data were included in both the pretraining and finetuning processes. Thereby, the MagBERT_DAPT_MagQA_Mixed and MagBERT_DAPT_MagQA models performed marginally worse than their non-DAPT counterparts, the MagBERT_MagQA_Mixed and MagBERT_MagQA models despite having been exposed to the evaluation contexts during pretraining (*cf*. Evaluation Metrics and Method), thus, corroborating that finetuning is more beneficial than DAPT. Our two highest performing BERT models were the MagBERT_MagQA and MagBERT_MagQA_Mixed models, with the latter having the best performance overall; this had been finetuned on both our MagQA data set, and SQuAD v2. This demonstrates that the extra training on generic English QA pairs has added benefit when finetuning domain-specific models for QA tasks.

When evaluated on the SQuAD v2 benchmark, poor performance was observed in BERT models that were not fine-tuned on SQuAD v2, i.e., the MagBERT_DAPT_MagQA and MagBERT_MagQA models, noting that the former even failed to answer a single answerable question correctly. BERT models that were finetuned on both SQuAD v2 and MagQA, MagBERT_DAPT_MagQA_Mixed and MagBERT_MagQA_Mixed models, showed considerably higher performance than BERT models which had exclusively been finetuned on our MagQA data set; but in contrast to our evaluation on the manually curated QA validation data set, BERT models trained on both data sets underperformed when compared to models that were only finetuned on SQuAD v2. Not surprisingly, our BERT_SQuADv2 model performed best of all BERT models that were evaluated on the SQuAD v2 benchmark. The MagBERT_DAPT_SQuADv2 model, which was further pretrained on the magnetic materials corpus, and solely finetuned on SQuAD v2 exhibited marginally worse performance. This indicates that when considering prompt-engineering queries for a BERT model whose context contains wholly generic English language, imparting knowledge from a specialized domain is not beneficial to the performance of the BERT model.

### BERT versus Larger BERT Model Architectures

Our realization of domain-specific BERT models for magnetism, using exclusively a finetuning process on vanilla RoBERTa-base and DeBERTa-base models, achieved comparable results to those of our MagBERT_MagQA_Mixed model, with all models displaying *F*1 scores and exact-match score that lie within 1.4% of each other. This implies that our workflow of autogenerating QA pairs from a structured material-property database can be extrapolated to larger BERT model architectures, while maintaining high performance of the language models on their downstream task of QA.

In stark contrast, when our larger BERT model architectures were tested on generic English language QA pairs, in the form of SQuAD v2, some interesting observations were made; *cf*. Technical Validation and Testing, where all of our MagBERT models that incorporated our MagQA data set in their finetuning stage of model training performed poorly on SQuAD v2 compared to our vanilla BERT_SQuADv2 model; while this same effect was not realized with the larger BERT models, RoBERTa-base and DeBERTa-base. The observed disparities can be explained by rationalizing the impact of the greater number of parameters that describe the vanilla RoBERTa and DeBERTa architectures. Thereby, it makes sense that a large and complex language, such as English, will need more parameters in a BERT model to gain a comprehensive linguistic understanding, even when domain-specific data is included in our finetuning process. By contrast, a highly specialized task that employs a much more compact vernacular, such as domain-specific material-property extraction, can be understood well when trained on this small domain. Indeed, our results show that the extra parameters of the RoBERTa and DeBERTa model architectures are not needed for a BERT model to understand the domain sufficiently. This results in a negligible difference in their performance against domain-specific QA tasks as observed, *i.e*. the performance of a domain-specific BERT model, when finetuned on domain-specific QA tasks, is not influenced by the size of the BERT model architecture.

### Ablation Studies to Understand QA Data Set Size Requirements

The results from our ablation study suggest that 10,000 domain-specific QA pairs is enough to finetune a high-performance domain-specific language model when paired with our defined train split of SQuAD v2. The 168,080 domain-specific QA pairs in the parent MagQA data set was predefined by the number of records in the magnetic-materials database from which they were extracted. Nonetheless, it is useful for researchers to know that this entire quantity is not needed to produce domain-specific BERT models for materials science that exhibit similar performance. In fact, our results suggest that more domain-specific QA pairs can result in a BERT model with slightly lower performance metrics, whereby our full MagQA data set afforded a BERT model with a lower *F*1 score and exact-match score than when the ablated data sets were used to finetune the same vanilla BERT-base model. We hypothesize that the provision of too many QA pairs could overfit a BERT model, resulting in its model weights learning the training data, rather than the semantic relationships that are present in each QA pair. Further to this point, the QA pairs autogenerated in this study comprise QA pairs that relate to Curie temperatures, Néel temperatures and their cognate material composition. This is a rather modest number of entities. However, we expect that as the number of entities used by the algorithms that autogenerate domain-specific QA pairs grows, the number of QA pairs that we need to realize high-performing domain-specific BERT models will grow with it.

### How Generalizable is Our Method to Realize Domain-Specific Language Models?

Up to this point, we have only presented the performance metrics of our own domain-specific language models that were finetuned via our herein described methodology. It is important to look at other studies that also employ the essence of this methodology, to see how our results compare. To this end, BERT models for the thermoelectric materials domain^14^ and the field of solar-cells^20^ have been created.

As per our technical evaluation, the thermoelectric BERT models were evaluated on a manually curated validation set of domain-specific QA pairs and their cognate multisentence contexts. Their algorithm generated 99,757 single-sentence QA pairs that included first-turn answerable and second-turn unanswerable questions, thereby providing a solid benchmark that can be compared against our study. In common with our results, the best performing thermoelectric BERT model (TE-BERT) on their manually curated validation set was a BERT model that had been finetuned on their domain-specific (TE-CDE-QA) data set that was mixed with SQuAD v2. Their study also found that finetuning the TE-BERT model solely on their domain-specific QA data set afforded a model with lower performance, while finetuning solely on SQuAD v2 achieved a BERT model that exhibited the worst performance when evaluated against their manually curated domain-specific QA data set. Table 12 shows a comparison of the performance metrics achieved by the TE-BERT and MagBERT_MagQA_Mixed models when tested against their respective domain-specific QA data sets.

**Table 12:** Performance Metric Achieved by the TE-BERT and MagBERT_MagQA_Mixed Models When Validated Against Their Respective Manually-Curated Domain-Specific QA Datasets

	Model	
Evaluation Metric	TE-BERT	MagBERT_MagQA_Mixed
*F*1 score	72.29%	78.43%
Exact match	67.93%	72.84%
HasAns *F*1 score	58.92%	68.53%
HasAns exact match	52.35%	60.14%
NoAns *F*1 score	98.80%	98.18%
NoAns exact match	98.80%	98.18%

As the MagBERT_MagQA_Mixed and TE-BERT models were finetuned and evaluated on different QA data sets, it is not fair to compare the metrics in Table 12 directly. Nonetheless, the trends presented do provide valuable insights into our study. Both models achieved high performance on domain-specific answerable questions and very high performance on unanswerable questions. Their similarities are also apparent through the concurrent trends in their ranked order of metrics and the ranked order of performance in training sets: i.e., their best performing model used their autogenerated QA data set mixed with SQuAD v2; their next best used just their autogenerated QA data set; then came the model that used just SQuAD v2. These consistent trends and similarities give robustness and validity to our workflow and offer an implicit veracity of the language models presented herein.

The aforementioned solar-cell BERT study by Li and Cole^20^ afforded a comprehensive set of 64 materials-domain-specific BERT models for solar cells. As with our study, Li and Cole autogenerated a solar-cell QA (SCQA) data set from a domain-specific database about materials and their cognate device properties. They then finetuned a comprehensive set of BERT model architectures that included BERT-base and BERT-large in both cased and uncased variations, either with or without DAPT, some of which were only finetuned on their SCQA data set, either with or without generic English language QA pairs included via the use of SQuAD v1.1.

There are some important methodological differences to note between our study, the TE-BERT model study^14^ and this solar-cell BERT^20^ modeling process. The algorithm used in the latter case to autogenerate the domain-specific SCQA data set searches the paper, from which the Q and A source information were sourced, for reference sentences that it thinks contains the relevant source information for generating the QA pairs. As the algorithm generates first and second-turn questions, it prioritizes a property value to be the answer, as opposed to the material. Thus, if the chosen reference sentence contains both the answer and the property specifier, a first-turn QA pair is generated that asks for the value of the property. If the reference sentence contains the material as well, a second turn QA pair is generated that asks for the type of material associated with the property value. If the sentences before or after the reference sentence do not contain the property specifier, then an unanswerable QA pair is generated. This algorithm generates a variety of QA types within the data set which should be noted, resulting in 42,882 first-turn, 4,386 second-turn and 1,212 unanswerable QA pairs.

Another difference is the way that BERT models for solar cells were evaluated. Instead of using a manually curated validation QA data set consisting of multisentence contexts, such as the QA data sets used to evaluate the MagBERT models in this study and the TE-BERT models,^14^ the SCQA data set used to evaluate the solar-cell BERT models was split into a train and validation split, whereby the train split consisted of 34,305 first-turn and 3,508 second-turn pairs, and the validation split consisted of 8,557 first-turn and 878 second-turn pairs. Notably, their study did not consider unanswerable questions. This is because they used the SQuAD v1.1 implementation that does not contain such questions, unlike the MagBERT and TE-BERT models which were both finetuned using SQuAD v2.

Despite these methodological differences to our workflow, some strong comparisons to the solar-cell BERT model study can still be made. As with the domain-specific families of MagBERT and TE-BERT models, domain-specific BERT models for solar cells exhibit the same pattern in their performance ranking when finetuned against manifold QA data set variations. The solar-cell BERT models finetuned on a mixed QA data set of SCQA and SQuAD v1.1 performed the best, the models finetuned solely on SQuAD v1.1 performed the worst, and the models finetuned solely on SCQA performed at an intermediate level, reiterating our claim that domain-specific BERT models can benefit from including generic English language QA pairs when finetuning them on domain-specific QA data sets.

The study showed that very little difference in performance was observed between domain-specific BERT models that had been finetuned on vanilla BERT-large and BERT-base model architectures, despite the BERT-large model having ∼340 million parameters, 24 layers and 16 attention heads compared to the BERT-base model which has ∼110 million parameters, 12 layers and 12 attention heads. The highest *F*1 scores and exact-match scores for the BERT-base model once finetuned on both the SCQA data set and SQuAD v1.1 were 76.726% and 73.685%, respectively. The corresponding domain-specific BERT-large model metrics were 76.929% and 74.245%, respectively. While these metrics are given for the highest performing BERT models, a total of 8 BERT-base models and 8 BERT-large models were finetuned on a mixed domain-specific and generic English language QA data set in the solar-cell study, and all *F*1 scores fell within 1% of each other, while all exact-match scores were contained within 2%. This agrees with our observations on finetuning the larger BERT architectures, RoBERTa and DeBERTa, using the MagQA data set, strongly reinforcing the case we make that our workflow can be generalized to larger BERT architectures while maintaining performance.

Our final comparison with the solar-cell BERT study relates to the effect of DAPT. The solar-cell BERT study reported on extensive work that was conducted to test the effect of DAPT, scraping a corpus of 161,183 published papers from the photovoltaics domain. This corpus was split into three data sets, scsmall (8,875 papers), scmedium (35,385 papers) and sclarge (161,183 papers). All permutations of BERT-base and BERT-large, cased and uncased, model architectures, that had been pretrained on scsmall, scmedium or sclarge corpora, were created before finetuning them on a mixed QA data set that comprises the SCQA data set and SQuAD v1.1. Table 13 lists the reference *F*1 scores and exact-match scores for the best performing domain-specific BERT-base and BERT-large model combinations in this solar-cell study, both with and without DAPT, when evaluated against their respective domain-specific QA data sets. Table 13 emphasizes that the performance of all domain-specific BERT-base and BERT-large models in the solar-cell study, that had been finetuned by the essence of our herein described methodology, lie within 1% of each other on *F*1 score, regardless of: whether or not they had been further pretrained; which size of pretraining data set had been used for DAPT (if any); or the size of the BERT-model architecture. This strongly reinforces our claims that DAPT is not needed when the essence of our herein described methodology can be applied to autogenerate large, high-quality, domain-specific QA data sets which are, in turn, employed to finetune vanilla BERT models to create high-performing domain-specific language models.

**Table 13:** Performance Metrics of BERT-Base and BERT-Large Models Both with and without DAPT in the Solar-Cell BERT study.^20^

	Evaluation metric			
Model	*F*1 score	Exact match	DAPT data set	Base model
bert-base-uncased-squadscqa1	76.349	72.415	None	BERT-base
bert-base-cased-sclarge-squadscqa1	76.816	73.301	sclarge	BERT-base
bert-large-uncased-squadscqa1	76.491	72.823	None	BERT-large
bert-large-uncased-scsmall-squadscqa1	76.997	73.907	scsmall	BERT-large

Overall, these MagQA, TE-BERT and solar-cell case studies demonstrate a clear, cross-domain pattern, thereby corroborating the notion that our workflow is robust and scientifically valid across the wider domain of materials science, including of course that of magnetism.

### Extractive versus Generative LLMs for Creating Domain-Specific QA Tasks about Materials Science

With the emergence and current popularity of large generative LMs, such as the Generative Pretrained transformer (GPT),^37,38,39^ Gemini^40–43^ and LLaMA^44–46^ model families, it is important to discuss them and explain why we chose not to use them for this study.

Generative LLMs have been used in materials-science applications that share some conceptual similarities with our workflow. For example, the MechGPT^47^ offers a mechanical materials pipeline that integrates a finetuned LLM for answering questions in the domain of mechanical materials. In common with the study presented herein, Buehler developed an autogenerated QA data set. This was used to finetune a OpenOrca-Platypus2–13B model, which is an open-weights generative language model based on the foundation Large Language Model Meta AI architecture, LLaMA 2–70b. In contrast, Buehler autogenerated a QA data set in a purely unsupervised manner, by applying a pdf version of his book, “Atomistic Modeling of Materials Failure”,^48^ to LLaMA-2–70b. The book was chunked into 500-character chunks, which were then passed to LLaMA-2–70b sequentially using a prompt to generate the QA pairs. While the autogenerated aspect of the finetuning data set is conceptionally similar to our method presented herein, it is important to note key differences in these methodologies. First, the unsupervised nature of the method used to autogenerate Buehler’s QA data set is distinct from our work. A typical concern with generative LLMs is that they can afford hallucinations, i.e., presenting outputs that can differ entirely from the objective with which they were originally prompted. However, the presented methodology uses grounded generation by including the chunk in-prompt to greatly reduce this risk. Second, no evaluation metrics for the finetuning QA data set reported by Buehler are presented for the MechGPT model. By stark contrast, our approach employs extractive algorithms (i.e., no new text is generated, thus removing the potential for hallucinations) combined with predefined question templates to generate our QA data set from an existing database that has already been manually evaluated with previously reported precision and recall scores; the error modes of our pipeline are therefore inherited from the source database rather than introduced during QA-pair generation (*cf.* the 72% precision reported in the Autogenerating a Large Domain-Specific Question-Answering Data Set section). This provides direct metrics to the quality of the QA pairs within our data set. Another difference is the model evaluation methodology. While it is expected that the exact procedures for evaluating generative versus extractive LLMs will differ, it should be noted that Buehler opted to evaluate the MechGPT model with multiple-choice questions for material-property extraction; whereas our models were evaluated by asking the model direct questions, thereby allowing our models to find the answer from within the context provided instead of choosing from a predefined list of candidates. The paper that introduces the MechGPT model does touch on this issue of uncertainties in generative LLM output by experimenting with “LLM temperature”. This is a parameter that can be passed to some generative LLMs to control the diversity of the output, whereby a higher temperature allows tokens with a lower probability to be selected in the generation process. However, this experiment was only conducted when prompting the trained MechGPT model in its evaluation stage, and it is not stated as a parameter that was used during the generation of the QA pairs used for finetuning.

Another domain-specific LLM is BioInspiredLLM, developed by Luu and Buehler.^49^ In common with the MechGPT model, BioInspiredLLM uses the chunking approach to pass chunks of text to a generative LLM (in this case LLaMA-2–13-chat) to distill QA pairs for a finetuning QA data set. The corpus used for distilling the QA pairs comprised of articles from Elsevier, Wiley, Springer Nature and the American Chemical Society in the biological materials domain. This QA data set was then used to finetune an Orca-2–13b model to form a conversational domain-specific LLM that is similar to that of MechGPT. Unlike the case of MechGPT, Luu and Buehler reported more extensive investigations on BioInspiredLLM, including the effect of finetuning Orca-2–13b on the cleaned text from the corpus itself; even though its results presented a lower degree of success compared with their “QA processing method” described above. This observation further highlights the importance of domain-specific data sets in the field of materials science. To this end, we note the importance of the LLM architectures for both the MechGPT model and BioInspiredLLM being classed as open-weights.

A marked advantage of the MechGPT model and BioInspiredLLM is that they can be described as “conversational LLMs”. The size and generalization of their parent foundation LLMs, combined with the generative capabilities from their autoregressive decoder architecture, allow them to generate text and keep track of conversations over thousands of tokens. This is showcased in the papers that describe the MechGPT model and BioInspiredLLM, whereby they demonstrate conversations with the models: prompting them to generate ideas for potential experiments and follow up studies, among other topics. Indeed, even at their most basic levels, these decoder-based model architectures can generate the next token in an output sequence based on the probability of that token being the correct token.

By contrast, encoder-only model architectures, such as BERT QA models, do not possess these kinds of capabilities, owing to the way that such models choose their output internally. Instead of generating new text, BERT models probabilistically predict the span of tokens in which the answer to the given question lies, within the span of the context in which it is given. This makes BERT models that have been fine-tuned on a QA task deliver an extractive rather than generative task. This has a distinct benefit in that BERT models can deliver domain-specific LLMs which offer trusted information extraction about materials-property relationships. This is because BERT models can only return information from the context which has been given for that specific question, thereby ensuring good precision and accuracy, while mitigating the hallucination problem that is witnessed in generative models.

Overall, these relative advantages and disadvantages of extractive versus generative domain-specific LLMs lead us to our preferred use of extractive language models. Notwithstanding that generative LLMs, such as those mentioned above, have all shown state-of-the-art performance in many NLP tasks, including QA and MLM; generative LLMs tend to operate as black-boxes and are either not open source, or do not disclose their training data. This makes it very difficult to ensure reproducibility and transparency. Furthermore, they deliver nondeterministic, nonidempotent pipelines and are infamous for suffering from hallucinations. We argue that domain-specific tasks such as materials-science extractive QA or CNER require precision and consistency in the output and form an idempotent, repeatable workflow. Moreover, security needs are a key issue for manifold industries. The scales of the most up-to-date LLMs have grown to the extent that generative LLMs are impossible to deploy locally on consumer-grade hardware, thereby making their utility dependent on providing user data to the cloud; *i.e*. necessitating the external release of data from an organization. This poses a substantial trust issue, even if security is assured by the cloud provider. Economic cost is a further consideration for industry: large-scale LLM providers market pay-per-token API schemes (e.g., OpenAI API), where a fee is charged per number of input and output tokens; such costs can easily become prohibitive from their cumulative use. The environmental cost of generative LLMs is also a 2-fold burden for industry. On the one hand, there is global concern about how the computational and data demands from widespread use of generative LLMs is contributing significantly to escalating energy costs and imposing critical sustainability issues.^50^ On the other hand, companies are increasingly being monitored and regulated against the sustainability targets of a nation or treaty organization (e.g., the United Nations Sustainable Development Goals).^51^

Notwithstanding these arguments, our MagQA data set could be beneficial to researchers exploring generative LLMs and agentic frameworks; for instance, its 168,080 context-grounded QA pairs could serve as a valuable resource for agentic frameworks. Thereby, potential use cases include: a knowledge base for RAG pipelines, whereby an agent retrieves domain-specific contexts to ground compositional, multistep answers about magnetic materials and their phase transition temperatures; a verification benchmark tool for agentic systems that must retrieve and cross-check materials data before responding; a finetuning data set for small specialist models, such as ours, that can be embedded as low-cost, deterministic extraction tools within multiagent pipelines, or even, smaller generative models such as those used in MechGPT and BioInspiredLLM. While we did not explore generative LLMs in our study, we have shown that small materials-domain-specific LLMs can be used as cost-effective, security assured alternatives that exhibit the high levels of data trustworthiness that are needed for myriad industries, especially those whose operations, innovation and growth opportunities stand to benefit from materials-science design and discovery pipelines.

## Conclusions

In summary, we have presented a study that showcases an algorithm to autogenerate a large, domain-specific data set of 168,080 QA pairs and demonstrate that it can be used to sufficiently alter the weights of BERT-base-cased models and larger architectures such as RoBERTa and DeBERTa to improve performance in the domain of magnetic materials. While our methodology yielded a data set size of 168,080 QA pairs, we show that similar or better performance can be achieved with significantly fewer QA pairs in our scientific domain of materials science.

Our work shows that imparting this domain-specific knowledge in the finetuning process of BERT model optimization is more important to model performance than imparting such knowledge via DAPT methods; although, should domain-specific finetuning QA data had not been available, competitive performance could have been achieved via the combination of DAPT and finetuning on a generic English language QA set, such as SQuAD v2. Nonetheless, it must be noted here that the compute time of the DAPT stage of BERT model optimization is up to ∼875 times more than that of the finetuning process. This calls into question the viability of DAPT methods, owing to their significantly higher cost and potential environmental impacts, noting that they deliver added gains of only up to ∼5% in this case study. All domain-adapted BERT models achieved competitive *F*1 and exact-match scores that were within ∼5% of each other. The MagBERT_MagQA_Mixed model, which performed best, was finetuned on both our autogenerated MagQA data set and SQuAD v2, achieving an *F*1 score of 78.43% and an exact-match score of 72.84%. This shows that the addition of generic English QA pairs, in the form of SQuAD v2, to a domain-specific QA fine-tuning process, can increase domain-specific BERT model performance on QA tasks.

When BERT models that were fine-tuned on our MagQA data set were evaluated on the SQuAD v2 benchmark, their performance was diminished. This indicates that, while domain-adaption can improve BERT model performance for the specified domain, these models should not be used for QA tasks in the context of generic English language; in such cases, one should use BERT models that have only been pretrained and finetuned on generic English language training data; indeed, as attention mappings show that the tokens in our domain-specific MagQA data set are not sufficient to adapt the models weights for the task of generic English language QA.

In broader terms, this work contributes to a growing portfolio of domain-specific BERT models for materials science that show high performance, when they have only been finetuned (i.e., not pretrained) using large, domain-specific QA data sets.^14,20^ This prospects a major opportunity to deliver high performing LLMs that are tailored for domain-specific prompt-engineering QA tasks, without the need for pretraining LLMs, i.e., one no longer needs computationally intensive resources to create LLMs for bespoke applications.

## Data Availability

The codebase used in this study to autogenerate the MagQA data set, the manually curated validation data set and train the models have been made publicly available on GitHub under the CC-BY-4.0 license.^52^ The seven models presented in this study have been uploaded to the Molecular Engineering Group area of HuggingFace under the CC-BY-4.0 license.^53^

## References

[1] Huang D., Cole J. M. (2025). Cost-Efficient Domain-Adaptive Pretraining of Language
Models for Optoelectronics Applications. J.
Chem. Inf. Model..

[2] Isazawa T., Cole J. M. (2024). How Beneficial Is
Pretraining on a Narrow Domain-Specific
Corpus for Information Extraction about Photocatalytic Water Splitting?. J. Chem. Inf. Model..

[3] Kumar P., Kabra S., Cole J. M. (2025). MechBERT:
Language Models for Extracting
Chemical and Property Relationships about Mechanical Stress and Strain. J. Chem. Inf. Model..

[4] Huang S., Cole J. M. (2022). BatteryBERT: A Pretrained
Language Model for Battery
Database Enhancement. J. Chem. Inf. Model..

[5] Zhao J., Huang S., Cole J. M. (2023). OpticalBERT and OpticalTable-SQA:
Text- and Table-Based Language Models for the Optical-Materials Domain. J. Chem. Inf. Model..

[6] Gupta T., Zaki M., Krishnan N. M. A., Mausam (2022). MatSciBERT:
A materials domain language
model for text mining and information extraction. Npj Comput. Mater..

[7] Lee J. (2020). BioBERT: a pre-trained biomedical language representation
model for
biomedical text mining. Bioinformatics.

[8] Beltagy, I. ; Lo, K. ; Cohan, A. SciBERT: A Pretrained Language Model for Scientific Text arXiv 2019 10.48550/arXiv.1903.10676

[9] Li, X. ; Yin, F. ; Sun, Z. ; Li, X. ; Yuan, A. ; Chai, D. ; Zhou, M. ; Li, J. Entity-Relation Extraction as Multi-Turn Question Answering arxiv 2019 10.48550/arXiv.1905.05529

[10] Jablonka K. M. (2023). 14 examples of how LLMs
can transform materials science and chemistry:
a reflection on a large language model hackathon. Digital Discovery.

[11] Dagdelen J. (2024). Structured information
extraction from scientific text with large
language models. Nat. Commun..

[12] Thway M. (2024). Harnessing GPT-3.5 for
text parsing in solid-state synthesis –
case study of ternary chalcogenides. Digital
Discovery.

[13] Polak M. P., Morgan D. (2024). Extracting accurate materials data from research papers
with conversational language models and prompt engineering. Nat. Commun..

[14] Sierepeklis O., Cole J. M. (2025). Autogenerating a Domain-Specific
Question-Answering
Data Set from a Thermoelectric Materials Database to Enable High-Performing
BERT Models. J. Chem. Inf. Model..

[15] Rajpurkar, P. ; Zhang, J. ; Lopyrev, K. ; Liang, P. SQuAD: 100,000+ Questions for Machine Comprehension of Text arXiv 2016. 10.48550/arXiv.1606.05250

[16] Gururangan, S. ; Marasović, A. ; Swayamdipta, S. ; Lo, K. ; Beltagy, I. ; Downey, D. ; Smith, N. A. Don’t Stop Pretraining: Adapt Language Models to Domains and Tasks. In Proceedings of the 58th annual meeting of the association for computational linguistics; ACL, 2020; pp. 8342-8360.

[17] Swain M. C., Cole J. M. (2016). ChemDataExtractor: A Toolkit for
Automated Extraction
of Chemical Information from the Scientific Literature. J. Chem. Inf. Model..

[18] Mavračić J., Court C. J., Isazawa T., Elliott S. R., Cole J. M. (2021). ChemDataExtractor
2.0: Autopopulated Ontologies for Materials Science. J. Chem. Inf. Model..

[19] Isazawa T., Cole J. M. (2022). Single Model for
Organic and Inorganic Chemical Named
Entity Recognition in ChemDataExtractor. J.
Chem. Inf. Model..

[20] Li Z., Cole J. M. (2025). Auto-generating
question-answering datasets with domain-specific
knowledge for language models in scientific tasks. Digital Discovery.

[21] Devlin, J. ; Chang, M.-W. ; Lee, K. ; Toutanova, K. BERT: Pre-training of Deep Bidirectional Transformers for Language Understanding. In Proceedings of the 2019 conference of the North American chapter of the association for computational linguistics: human language technologies; ACL, 2018.

[22] Wolf, T. ; Debut, L. ; Sanh, V. ; Chaumond, J. ; Delangue, C. ; Moi, A. ; Cistac, P. ; Rault, T. ; Louf, R. ; Funtowicz, M. HuggingFace’s Transformers: State-of-the-art Natural Language Processing. In Proceedings of the 2020 conference on empirical methods in natural language processing: system demonstrations; ACL, 2020.

[23] Rasley, J. ; Rajbhandari, S. ; Ruwase, O. ; He, Y. August. Deepspeed: System optimizations enable training deep learning models with over 100 billion parameters. In Proceedings of the 26th ACM SIGKDD international conference on knowledge discovery & data mining; ACM, 2020; pp. 3505–3506. DOI: 10.1145/3394486.3406703.

[24] Rajpurkar, P. ; Jia, R. ; Liang, P. Know What You Don’t Know: Unanswerable Questions for SQuAD. In Proceedings of the 56th Annual Meeting of the Association for Computational Linguistics; ACL 2018; pp. 784-789.

[25] Zhao J., Cole J. M. (2022). A database of refractive indices and dielectric constants
auto-generated using ChemDataExtractor. Sci.
Data.

[26] Sierepeklis O., Cole J. M. (2022). A thermoelectric
materials database auto-generated
from the scientific literature using ChemDataExtractor. Sci. Data.

[27] Huang S., Cole J. M. (2020). A database of battery materials auto-generated
using
ChemDataExtractor. Sci. Data.

[28] Isazawa T., Cole J. M. (2023). Automated Construction
of a Photocatalysis Dataset
for Water-Splitting Applications. Sci. Data.

[29] Huang D., Cole J. M. (2024). A database of thermally
activated delayed fluorescent
molecules auto-generated from scientific literature with ChemDataExtractor. Sci. Data.

[30] Dong Q., Cole J. M. (2022). Auto-generated database of semiconductor
band gaps
using ChemDataExtractor. Sci. Data.

[31] Kumar P., Kabra S., Cole J. M. (2024). A Database
of Stress-Strain Properties
Auto-generated from the Scientific Literature using ChemDataExtractor. Sci. Data.

[32] Kelly C. R., Cole J. M. (2025). A dataset of Curie and Néel
temperatures auto-generated
with ChemDataExtractor and the Snowball algorithm. Sci. Data.

[33] Dong Q., Cole J. M. (2023). Snowball 2.0: Generic
Material Data Parser for ChemDataExtractor. J. Chem. Inf. Model..

[34] Agichtein, E. ; Gravano, L. Snowball: Extracting relations from large plain-text collections Proceedings of the fifth ACM conference on Digital libraries; ACM, 2000; pp. 85–94.

[35] Kumar, P. QA-Annotator. GitHub repository. https://github.com/gh-PankajKumar/QA-Annotator.

[36] Shi Y. (2021). Performances variations
of BiFeO_3_-based ceramics induced
by additives with diverse phase structures. CrystEngComm..

[37] Brown, T. ; Mann, B. ; Ryder, N. ; Subbiah, M. ; Kaplan, J.D. ; Dhariwal, P. ; Neelakantan, A. ; Shyam, P. ; Sastry, G. ; Askell, A. Language Models are Few-Shot Learners arXiv 2020. 10.48550/arXiv.2005.14165

[38] Achiam, J. ; Adler, S. ; Agarwal, S. ; Ahmad, L. ; Akkaya, I. ; Aleman, F.L. ; Almeida, D. ; Altenschmidt, J. ; Altman, S. ; Anadkat, S. GPT-4 Technical Report arXiv 2024. 10.48550/arXiv.2303.08774

[39] Singh, A. ; Fry, A. ; Perelman, A. ; Tart, A. ; Ganesh, A. ; El-Kishky, A. ; McLaughlin, A. ; Low, A. ; Ostrow, A.J. ; Ananthram, A. OpenAI GPT-5 System Card arXiv 2025. 10.48550/arXiv.2601.03267

[40] Team, G. ; Anil, R. ; Borgeaud, S. ; Alayrac, J.B. ; Yu, J. ; Soricut, R. ; Schalkwyk, J. ; Dai, A.M. ; Hauth, A. ; Millican, K. Gemini: A Family of Highly Capable Multimodal Models arXiv 2025. 10.48550/arXiv.2312.11805

[41] Team, G. ; Georgiev, P. ; Lei, V.I. ; Burnell, R. ; Bai, L. ; Gulati, A. ; Tanzer, G. ; Vincent, D. ; Pan, Z. ; Wang, S. Gemini 1.5: Unlocking multimodal understanding across millions of tokens of context arXiv 2024. 10.48550/arXiv.2403.05530

[42] Comanici, G. ; Bieber, E. ; Schaekermann, M. ; Pasupat, I. ; Sachdeva, N. ; Dhillon, I. ; Blistein, M. ; Ram, O. ; Zhang, D. ; Rosen, E. Gemini 2.5: Pushing the Frontier with Advanced Reasoning, Multimodality, Long Context, and Next Generation Agentic Capabilities arxiv 2025, http://arxiv.org/abs/2507.06261.

[43] Gemini 3.1 Pro. https://deepmind.google/models/model-cards/gemini-3-1-pro/.

[44] Touvron, H. ; Lavril, T. ; Izacard, G. ; Martinet, X. ; Lachaux, M.A. ; Lacroix, T. ; Rozière, B. ; Goyal, N. ; Hambro, E. ; Azhar, F. , LLaMA: Open and Efficient Foundation Language Models. arXiv 2023 10.48550/arXiv.2302.13971

[45] Touvron, H. ; Martin, L. ; Stone, K. ; Albert, P. ; Almahairi, A. ; Babaei, Y. ; Bashlykov, N. ; Batra, S. ; Bhargava, P. ; Bhosale, S. ; Bikel, D. Llama 2: Open Foundation and Fine-Tuned Chat Models arXiv 2023. 10.48550/arXiv.2307.09288

[46] Grattafiori, A. ; Dubey, A. ; Jauhri, A. ; Pandey, A. ; Kadian, A. ; Al-Dahle, A. ; Letman, A. ; Mathur, A. ; Schelten, A. ; Vaughan, A. ; Yang, A. The Llama 3 Herd of Models arXiv 2024. 10.48550/arXiv.2407.21783

[47] Buehler M. J. (2024). MechGPT,
a Language-Based Strategy for Mechanics and Materials Modeling That
Connects Knowledge Across Scales, Disciplines, and Modalities. Appl. Mech. Rev..

[48] Atomistic Modeling of Materials Failure; Buehler, M. J. eds., Springer US: Boston, MA; 2008. 10.1007/978-0-387-76426-9

[49] Luu, R. K. ; Buehler, M. J. BioinspiredLLM: Conversational Large Language Model for the Mechanics of Biological and Bio-inspired Materials arXiv 2023. 10.48550/arXiv.2309.08788 PMC10933662 38145334

[50] Sandonas, L. M. ; Balcells, D. ; Bochkarev, A. ; Cole, J.M. ; Deringer, V.L. ; Dobrautz, W. ; Ehrenhofer, A. ; Frank, T. ; Friederich, P. ; Friedrich, R. Perspective: Towards sustainable exploration of chemical spaces with machine learning arXiv 2026. 10.48550/arXiv.2604.00069

[51] UN General Assembly Transforming our world: the 2030 Agenda for Sustainable Development; UN General Assembly. https://www.refworld.org/legal/resolution/unga/2015/en/111816.

[52] Kelly, C. R. MagBERT_from_Curie_Neel_Dataset. https://github.com/C-R-Kelly/MagBERT_from_Curie_Neel_Dataset.

[53] MagBERT Models. https://huggingface.co/collections/CambridgeMolecularEngineering/magbert-models.

